# Inflammatory priming enhances mesenchymal stromal cell secretome potential as a clinical product for regenerative medicine approaches through secreted factors and EV-miRNAs: the example of joint disease

**DOI:** 10.1186/s13287-020-01677-9

**Published:** 2020-04-28

**Authors:** Enrico Ragni, Carlotta Perucca Orfei, Paola De Luca, Carlotta Mondadori, Marco Viganò, Alessandra Colombini, Laura de Girolamo

**Affiliations:** 1 IRCCS Istituto Ortopedico Galeazzi, Laboratorio di Biotecnologie Applicate all’Ortopedia, Via R. Galeazzi 4, Milan, 20161 Italy; 2 IRCCS Istituto Ortopedico Galeazzi, Cell and Tissue Engineering Laboratory, Via R. Galeazzi 4, Milan, 20161 Italy

**Keywords:** Mesenchymal stromal cells, Adipose tissue, Secreted factors, miRNAs, Secretome, Osteoarthritis, Inflammation, Cartilage, Macrophage

## Abstract

**Background:**

Mesenchymal stromal cell (MSC)-enriched products showed positive clinical outcomes in regenerative medicine, where tissue restoration and inflammation control are needed. GMP-expanded MSCs displayed an even higher potential due to exclusive secretion of therapeutic factors, both free and conveyed within extracellular vesicles (EVs), collectively termed secretome. Moreover, priming with biochemical cues may influence the portfolio and biological activities of MSC-derived factors. For these reasons, the use of naive or primed secretome gained attention as a cell-free therapeutic option. Albeit, at present, a homogenous and comprehensive secretome fingerprint is still missing. Therefore, the aim of this work was to deeply characterize adipose-derived MSC (ASC)-secreted factors and EV-miRNAs, and their modulation after IFNγ preconditioning. The crucial influence of the target pathology or cell type was also scored in osteoarthritis to evaluate disease-driven potency.

**Methods:**

ASCs were isolated from four donors and cultured with and without IFNγ. Two-hundred secreted factors were assayed by ELISA. ASC-EVs were isolated by ultracentrifugation and validated by flow cytometry, transmission electron microscopy, and nanoparticle tracking analysis. miRNome was deciphered by high-throughput screening. Bioinformatics was used to predict the modulatory effect of secreted molecules on pathologic cartilage and synovial macrophages based on public datasets. Models of inflammation for both macrophages and chondrocytes were used to test by flow cytometry the secretome anti-inflammatory potency.

**Results:**

Data showed that more than 60 cytokines/chemokines could be identified at varying levels of intensity in all samples. The vast majority of factors are involved in extracellular matrix remodeling, and chemotaxis or motility of inflammatory cells. IFNγ is able to further increase the capacity of the secretome to stimulate cell migration signals. Moreover, more than 240 miRNAs were found in ASC-EVs. Sixty miRNAs accounted for > 95% of the genetic message that resulted to be chondro-protective and M2 macrophage polarizing. Inflammation tipped the balance towards a more pronounced tissue regenerative and anti-inflammatory phenotype. In silico data were confirmed on inflamed macrophages and chondrocytes, with secretome being able to increase M2 phenotype marker CD163 and reduce the chondrocyte inflammation marker VCAM1, respectively. IFNγ priming further enhanced secretome anti-inflammatory potency.

**Conclusions:**

Given the portfolio of soluble factors and EV-miRNAs, ASC secretome showed a marked capacity to stimulate cell motility and modulate inflammatory and degenerative processes. Preconditioning is able to increase this ability, suggesting inflammatory priming as an effective strategy to obtain a more potent clinical product which use should always be driven by the molecular mark of the target pathology.

## Background

At present, few mesenchymal stromal cell (MSC)-based products have gained marketing approval by the regulatory authorities [1] and mainly rely on MSCs derived from bone marrow (BMMSCs), adipose tissue (ASCs), or umbilical cord (WJMSCs), reflecting the main sources of MSCs tested for clinical trials being currently bone marrow, adipose, and umbilical cord tissues [2]. Clinical indications are largely related to musculoskeletal (bone regeneration and osteoarthritis (OA)) and inflammatory (Chron’s and GvHD) diseases. This is in agreement with positive clinical outcomes of MSC-enriched products, as bone marrow concentrate or microfragmented fat tissue, in one-step regenerative medicine approaches where both tissue restoration and inflammation control are needed, as in joint pathologies [3]. Under these premises, due to higher concentration of the active biological components, GMP-expanded MSCs are postulated to have an even higher and more focused potential in both autologous and allogeneic therapies. Nevertheless, available products are all sourced and manufactured through distinct processes, rendering direct potency comparison and disease-driven selection challenging. Moreover, sheer complexity of living mammalian cells makes MSC characterization/specification at both functional and molecular levels extraordinarily difficult, delaying a fast and wider use of these cells in regenerative medicine applications.

These premises prompted the identification of a MSC-based product able to retain originating cell potency but easier to be characterized and standardized. In the last years, it has been proposed that therapeutic effects of MSCs may be ascribed to secreted cytokines and growth factors [4, 5], as well as different types of extracellular vesicles (EVs) [6], altogether defining the secretome. EVs shuttle both 3′UTR mRNA fragments competing with recipient miRNAs or proteins [7], together with mRNAs and miRNAs with multiple functions as immunomodulation and tissue restoration [8, 9]. Thus, secretome as a whole or its separate components gained attention as an innovative cell-free medicinal product for those pathologies involving both immune system and tissue homeostasis unbalance [10, 11].

With the view of MSC secretome clinical translation, to date, few phase I/II clinical trials were performed [12–16]. Together with safety, secretome showed reduction of inflammation and long-lasting clinical improvements [13, 16]. For these reasons, attention increased, and at present (December 2019), 4 trials are registered for MSC secretome or purified EVs in a wide range of pathologies: refractory macular holes (NCT03437759), type 1 diabetes mellitus (NCT02138331), chronic skin ulcer (NCT04134676), and acute ischemic stroke (NCT03384433). Positive results will stimulate further studies to test safety and efficacy in other diseases where MSCs have been studied in completed phase I/II/III clinical trials, as for the treatment of musculoskeletal diseases where positive outcomes laid the foundations for the marketing of MSC-based products [17].

MSCs and secretome potential may be further modulated by extrinsic factors, such as tissue source or pro-/anti-inflammatory environment [18, 19], able to polarize MSCs towards either a pro-inflammatory or an immuno-suppressive phenotype [20], with pro-inflammatory factors such as TNFα, IL-1β, or IFNγ activating the latter that is also involved in tissue regeneration [11]. In particular, IFNγ results in the differential expression of 210 cellular proteins [21], including immunomodulatory molecules [22]. Consistently, in an equine model of osteoarthritis, IFNγ-primed MSCs reduced synovial effusion, improved cartilage gross appearance, and delayed progression of proteoglycan loss [23]. Similarly, in experimental colitis, IFNγ-preconditioned MSCs showed a significant reduction of inflammatory responses [24]. In general, the majority of tested priming approaches, through modulation of both secreted factors and EV-embedded miRNAs [25], were able to improve MSC therapeutic efficacy [26] and laid the groundwork for a higher clinical efficacy of the secretome.

Nevertheless, many limitations still delay the clinical translation, such as high costs, variable effects depending on tissue source and donor variability, and lack of clear therapeutic application based on the portfolio of secreted molecules. Further, to facilitate approval of therapeutic applications, xenogenic components such as FBS or inflammatory molecules used for priming should be avoided at least during the EV production and harvest phase. Notably, the majority of reports assessing both the secreted factors and their modulation after priming do not consider this aspect, with exogenous molecules and particles possibly diluting, altering, or blocking some effects of MSC secretome. Therefore, the aims of this study are to characterize both secreted factors and EV-embedded miRNAs and to evaluate their modulation after IFNγ preconditioning, in the serum-free secretome of ASCs. Bioinformatics analysis of potentially regulated pathways and a list of markers for the development of future release assays are provided, in order to allow a more profound understanding of MSC secretome for the treatment of different conditions such as OA as an example of pathology-focused potency evaluation.

## Methods

### Adipose-derived mesenchymal stromal cell isolation and expansion

Adipose waste material from four female donors (median 54 years old, min 45, max 61) undergoing liposuction was digested with 0.075% w/v type I collagenase (30 min at 37 °C) (Worthington Biochemical Co, Lakewood, NJ, USA). Digested samples were filtered through a cell strainer and centrifuged (1000×*g*, 5 min). Pelleted cells were seeded at 5 × 10^3^ cells/cm^2^ in DMEM + 10% FBS (GE Healthcare, Piscataway, NJ, USA) and penicillin-streptomycin (Life Technology, Carlsbad, CA, USA). Cells were cultured at 37 °C, 5% CO_2_, and 95% humidity. Cells were cultured until 90% confluence with medium change each 3 days. At 90% density, cells were detached and either frozen or seeded at 4000 cells/cm^2^. Cells were used for experiments at passage 5 at 90% confluence, 1 day after the last medium change. ASCs were primed with 10 ng/ml IFNγ for 48 h (iASCs). Afterwards, culture flasks were washed five times with PBS and medium without serum added. After 48 h, conditioned medium (secretome) was collected and further processed.

### ASC characterization

Flow cytometry was used to score positive or negative MSC or hemato/endothelial markers (CD44-PE Vio770 clone REA690, CD73-PE clone REA804, and CD90-FITC clone REA897 or CD34-FITC clone AC136, CD31-PerCp Vio700 clone REA730, and CD45-PE Vio770 clone REA747; Miltenyi Biotec, Bergisch Gladbach, Germany) with a CytoFLEX flow cytometer (Beckman Coulter, Fullerton, CA, USA) collecting a minimum of 10,000 events.

### qRT-PCR analysis

ASCs in growth medium or growth medium supplemented with 10 ng/ml IFNγ for 48 h were washed twice with PBS and directly lysed in TRIzol reagent (Sigma-Aldrich, St. Louis, MO, USA). RNA was extracted following standard procedures [27]. First-strand cDNAs were synthesized with iScript cDNA synthesis kit (Bio-Rad Laboratories, CA, USA) [28]. Primers for *CXCL9/10*, *CCL5/8*, *COX2*, *HGF*, *HIF1A*, *IDO1*, *IL-6*, *IL-8*, and *FGF2* were designed using the NCBI Primer Designing Tool (http://www.ncbi.nlm.nih.gov/tools/primer-blast/). *TBP* was used as a reference for gene quantification. Primer sequences will be provided upon request. Quantifications were performed using “PowerUp SYBR Green Master Mix” (Applied Biosystems, Warrington, UK) and Comparative Ct Method in a StepOne Plus PCR Real Time Instrument (Applied Biosystems) [29]. Unprimed ASCs were used as control.

### Extracellular vesicle isolation and characterization

Conditioned medium was collected and subjected to differential centrifugation steps to remove broken cells and debris. Briefly, the medium was centrifuged at 4 °C for 15 min at 1000×*g* and 2000×*g* and twice at 4000×*g*. Five milliliters of last supernatant was 1:2 diluted with PBS and centrifuged at 100,000×*g* for 9 h at 4 °C in a 70Ti rotor (Beckman Coulter, Fullerton, CA, USA), and EV pellets were processed as follows:

i) Flow cytometry: before ultracentrifugation, conditioned media were supplemented with 10 μM CFSE (Sigma-Aldrich) and incubated for 1 h at 37 °C. After ultracentrifugation, as previously described, pellets were suspended in 100 μl PBS per 10 ml of processed medium. Labeled EVs were 1:10,000 diluted in PBS and 100 μl stained with anti CD81-APC clone 5A6 and anti CD63-APC clone H5C6 (Biolegend, San Diego, CA, USA) for 30 min at 4 °C in the dark. Antibodies were used individually. Collection was performed with a CytoFLEX flow cytometer collecting events for 30 s at 10 μl/min flow rate. Flow cytometer was set with a reference bead mix (Biocytex, Marseille, France) composed of a FITC fluorescent mixture of spheres (100 nm, 300 nm, 500 nm, and 900 nm). Gains were FSC = 106, SSC = 61, FITC = 272, PE = 116, and PC7 = 371. FITC threshold was set at 500 to include 100-nm beads and some smaller debris in the FITC channel.
ii) Transmission electron microscopy: after EV pellet suspension in PBS, 5 μl was absorbed on formvar carbon-coated grids for 10 min. Drops were blotted with filter paper. Two percent uranyl acetate aqueous suspension was used to negative stain for 10 min, and excess was removed by filter paper. Afterwards, the grid was dried at room temperature. Samples were examined with a TALOS L120C transmission electron microscope (Thermo Fisher Scientific, Waltham, MA, USA) at 120 kV.
iii) Nanoparticle tracking analysis (NTA): EVs in conditioned medium (1:3 diluted in PBS) or purified EVs in PBS (1:100 diluted) were visualized by the NanoSight LM10-HS system (NanoSight Ltd., Amesbury, UK). Three recordings of 30 s were performed for each sample. Collected data were analyzed by the NTA software, providing concentration measurements and high-resolution particle size distribution profiles.

### Screening of EV-embedded miRNA expression

EV pellets were dissolved in TRIzol reagent and RNA extracted with the miRNeasy Kit and RNeasy CleanUp Kit (Qiagen, Hilden, Germany), following the manufacturer’s instruction. During extraction, 6 pg of a non-human synthetic miRNA (*Arabidopsis thaliana* ath-miR-159a) was added to each sample as a spike-in to monitor the technical variability during the isolation and following reactions for eventual equalization of panels A and B of the OpenArray® platform (Life Technologies). cDNAs were prepared by standard reverse transcription (RT) and preamplification procedures with A and B independent kits, followed by real-time RT-PCR analysis with the QuantStudio™ 12 K Flex OpenArray® Platform (QS12KFlex) as previously described [30]. The Gene Expression Suite Software (Life Technologies) was used to process miRNA expression data from the A and B miRNA panels, together covering 754 well-characterized human miRNA sequences from the Sanger miRBase v21. The global mean was selected as the normalization method due to the high correlation between samples [31]. C_RT_ > 28 were considered as unamplified. Normalized miRNA expression was determined using the relative quantification 2 − ΔC_RT_. Values are shown as normalized Ct and ratios calculated separately for each sample as primed vs unprimed and then mean values ± SD calculated.

### ELISA assays

Concentrations of 200 soluble inflammatory and growth factors, chemokines, receptors, and cytokines in conditioned cell culture medium were determined by Quantibody® Human Cytokine Array 4000 Kit (https://www.raybiotech.com/quantibody-human-cytokine-array-4000/) according to the manufacturers’ instructions (RayBiotech, Norcross, GA, USA). 1:2 dilutions of culture supernatants were made to have absorbance readings within the standard curve values. Only factors that were detected above single assay threshold in all samples, either resting or primed, were selected for the analysis. The amount of each factor was calculated multiplying the concentration in pg/ml per the volume of culture medium and eventually divided per million cell to obtain a pg/10^6^ cell value. Values are shown as pg or ng per million cells and ratios calculated separately for each sample as primed vs unprimed and then mean values ± SD calculated.

### Construction and analysis of protein-protein interaction (PPI) networks

The online tool STRING (http://www.string-db.org) to construct interactome maps of ELISA identified proteins (STRING database v11 data accessed: March 2020). The indicated network properties include organism, *Homo sapiens*; meaning of network edges, evidence; active interaction sources, experiments and databases; and minimum required interaction scores, medium confidence (0.400).

### Pathway analysis

#### Identification of functional annotations

##### Protein

Proteins identified in ASCs or iASC secretome were subjected to functional enrichment analysis to provide insight into the functional associations of these protein subsets. This analysis was performed using GO:TermFinder for enrichment of biological process (BP) Gene Ontology (GO) terms (https://go.princeton.edu/cgi-bin/GOTermFinder). Statistical significance was calculated setting *p* value cutoff for significant shared GO terms at 0.01 and using Bonferroni and FDR corrections [32]. When no enriched GO terms were found, a list of proteins was submitted to the PANTHER web interface (http://www.pantherdb.org/) to identify proteins encompassing the same functional classifications, following default settings [33]. Selected classification was Panther-GO Slim Biological Process.

##### miRNA

The predicted miRNA targets were annotated into functional BP using DIANA-miRPath V. 3 (http://snf-515788.vm.okeanos.grnet.gr/), using microT-CDS to score for predicted miRNA-mRNA interaction (threshold 0.8), *p* value threshold of 0.05 for the GO category and FDR correction [34]. Further analysis was performed using the microRNA Target Filter tool in Ingenuity Pathway Analysis (IPA; Ingenuity® Systems, www.ingenuity.com). Filters were confidence “experimentally observed” and disease “skeletal and muscular disorders” or “inflammatory response” to score focused pathways (see the “Results” section).

#### Principal component analysis

Principal component analysis (PCA) plots were generated scoring factors or miRNAs with ClustVis package (https://biit.cs.ut.ee/clustvis/), after row centering [35]. pg factors per million cells or miRNA C_RT_ after normalization values were used.

#### Secretome validation on inflamed macrophages and chondrocytes by flow cytometry

Human primary monocytes were isolated from three buffy coats of healthy donors that were obtained from the local blood bank, by Ficoll (GE Healthcare, Little Chalfont, UK) density gradient separation, followed by positive magnetic selection using CD14 microbeads (MACS, Miltenyi) [36]. After isolation, CD14+ monocytes were plated in 12-well plates at a density of 300,000 monocytes/cm^2^ and cultured for 5 days in complete medium supplemented with 10% heat-inactivated FBS and 1% penicillin-streptomycin-glutamine. Macrophage colony-stimulating factor (M-CSF, Peprotech Inc., Rocky Hill, NJ, USA) was added to the culture medium at 20 ng/ml to differentiate monocytes towards macrophages [37]. Following differentiation into macrophages for 5 days, cells were then cultured for 24 h in complete medium (supplemented with 10% ultracentrifuged FBS to avoid serum EVs) in 4 different conditions. Experiments were conducted either in the absence or in the presence of pro-inflammatory cytokines, IFNγ at 100 ng/ml and TNFα at 100 ng/ml, always including M-CSF at 20 ng/ml (human, all from Peprotech) [37]. Specifically, macrophages cultured only with M-CSF represented the unstimulated macrophages (M0), whereas macrophages cultured with IFNγ and TNFα represented the polarized macrophages (M1). These two conditions were used as control groups. Other two conditions were prepared to test the anti-inflammatory potential of secretome on M1 macrophages in the presence of an inflammatory stimulus. To this aim, M1 macrophages cultured with M-CSF, IFNγ, and TNFα were treated with pooled secretome obtained by resting ASC or by ASC primed with IFNγ. All the procedures were conducted using 10% ultracentrifuged FBS as a medium supplement. After 24 h, cells were washed twice with PBS and detached by incubating them with cell dissociation buffer (Thermo Fisher, Frankfurt, Germany) for 10 min. Cells were then centrifuged at 400×*g* for 7 min, suspended in FACS buffer and counted. Fifty thousand cells were then stained with monoclonal antibodies to analyze through flow cytometry technique the expression of M1 pro-inflammatory (CD86) and M2 anti-inflammatory (CD163) macrophage markers. Briefly, cells were stained at 4 °C for 30 min in the dark with anti-human CD14-FITC (clone TUK4, Miltenyi), for macrophage gating; anti-human CD86-PE (Clone FM95, Miltenyi), for M1 phenoptype; and anti-human CD163-PE-Vio770 (Clone REA812, Miltenyi), for M2 phenotype. Unstained cells were used as negative control for fluorescence. After wash in FACS buffer, at least 30,000 events were acquired with a Cytoflex flow cytometer (Beckman Coulter).

Chondrocytes were obtained from three OA (Kellgren Lawrence III or IV) patients undergoing total hip arthroplasty, as in [38]. Briefly, the cartilage was harvested with a scalpel from non-weight bearing superficial areas of femoral head/neck, and chondrocytes were isolated by enzymatic digestion (37 °C, 22 h) with 0.15% w/v type II collagenase (Worthington Biochemical, Lakewood, NJ, USA), then cultured in DMEM + 10% FBS. When 90% confluence was reached, cells were detached and seeded at 4000 cells/cm^2^. At passage 3, chondrocytes were seeded and, when at 90% confluence, either control medium or control medium + 25 pg/ml IL-1β added. After 3 days, four conditions were run: control (DMEM + 10% FBS), 25 pg/ml IL-1β in control medium, IL-1β in resting ASC secretome (+ 10% FBS), or IL-1β in primed ASC secretome (+ 10% FBS). Ultracentrifuged FBS was used to avoid serum EVs interference. After 4 days, cells were washed, detached, and counted. Fifty thousand cells were stained in the dark at 4 °C for 30 min with human anti-VCAM1 (clone HA58, Miltenyi). Unstained cells were used as negative control for fluorescence. After FACS buffer wash, at least 30,000 events were acquired with a Cytoflex flow cyotometer (Beckman Coulter).

#### Statistical analyses

For secretome analysis, in each experiment, four independent cultures were included. Only proteins or miRNAs present and quantified in all unprimed, primed, or both conditions were considered as positively identified. Statistical analysis was performed using the GraphPad Prism software (GraphPad, San Diego, CA, USA). Kolmogorov-Smirnov normality test was used to test normal data distribution. Grubbs’ test was used to identify outliers. A one sample *t* test was used to compare the mean ratios (IFNγ vs untreated, factors, or miRNAs) with a hypothetical mean value set as 1 as per absence of modulation. The level of significance was set at *p* value value < 0.05. Data was presented as mean ± SD. For macrophages and chondrocytes analysis, three independent populations were studied. Kolmogorov-Smirnov and Grubb’s tests were used. One sample *t* test was used for ratios to compare means vs reference values set as 1, and *t* test of two means was used for other comparisons, with significance set at *p* value < 0.05. Data was presented as mean ± SD.

## Results

### Effect of the stimulation with IFNγ on ASCs

**Fig. 1:**
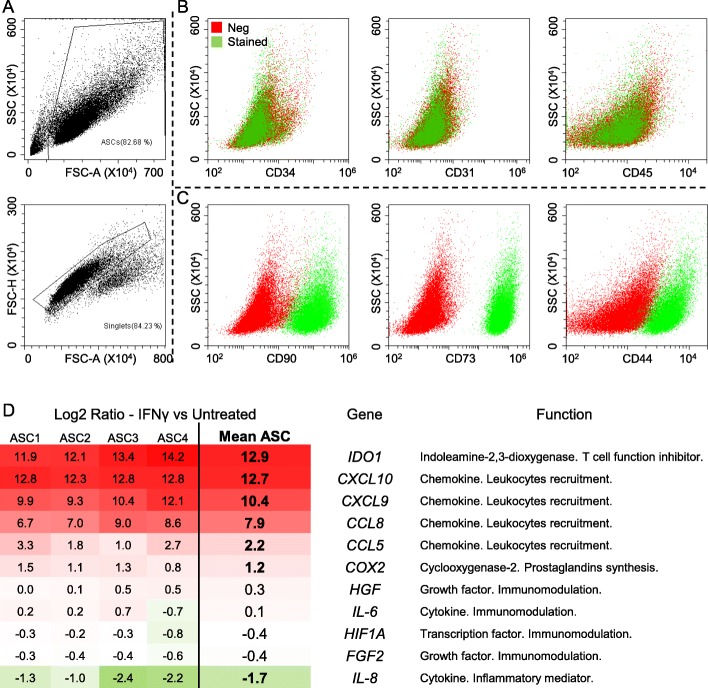
ASC characterization and inflammatory modulation. **a**, **c** Representative dot plots of hemato-endothelial (CD31-34-45) and MSC (CD44-73-90) markers in ASCs. One representative cell isolate is shown. **d** IFNγ effects on targets reported to be modulated by different inflammatory stimuli. Log2 ratios of iASCs vs ASCs are shown

ASCs were tested using specific surface markers by flow cytometry: ASCs (Fig. 1a) were completely negative for the hemato-endothelial markers (CD31, CD34, and CD45; Fig. 1b) and > 95% positive for the mesenchymal stromal cells markers CD44, CD73, and CD90 (Fig. 1c). To determine whether inflammatory stimuli may influence surface marker expression, cells were tested in the presence of IFNγ at a concentration of 10 ng/ml for 48 h. No alterations were detected (data not shown). Cell viability was also not affected, with 96% ± 0.5 viable cells before and 95% ± 1 after IFNγ stimulation.

We next examined the effect of IFNγ on the RNA expression of several reporters known to be related with the transcriptional response to different inflammatory stimuli (Fig. 1d). IFNγ response trend was shared across all ASCs under study suggesting a conserved pattern of modulation, with significant (fold < or > 2, *p* value < 0.05) increase for *IDO1*, *CXCL9*/*10*, *CCL5*/*8*, and *COX2* and decrease for *IL8* (Fig. 1d). Notably, with the only exception of CCL5 (fold of 1.12 ± 1.25), CXCL9/10, CCL8, and IL8 confirmed the significant modulation also when their presence was monitored in the conditioned medium after 48 h removal of the inflammatory stimulus (see next paragraphs).

### ASC-secreted factors

**Fig. 2:**
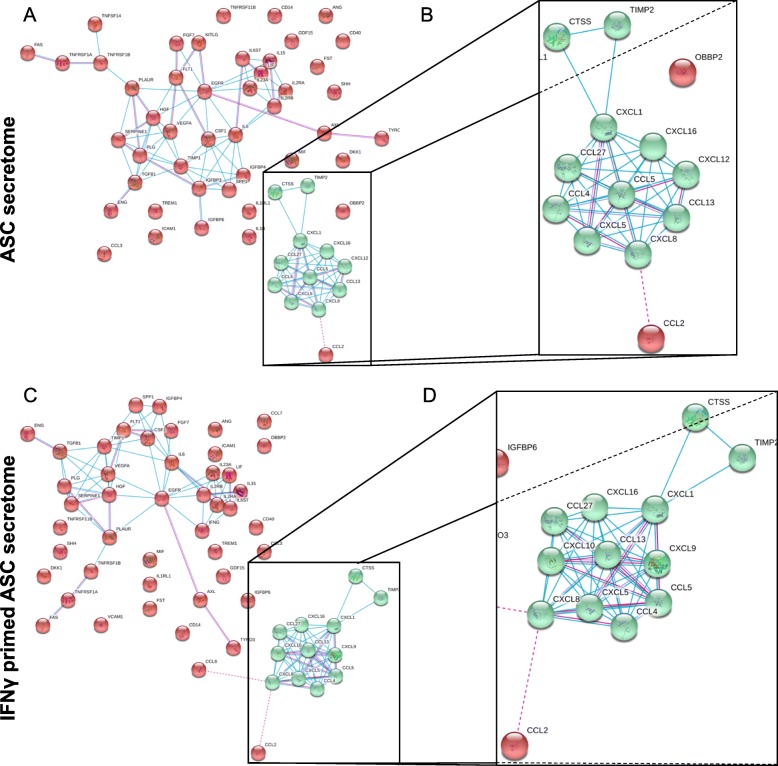
Functional association network for identified secreted factors. **a**, **b** Using the online tool STRING, protein-protein interaction (PPI) levels for 57 proteins of the resting ASC secretome were mined. **c**, **d** PPI levels for the 58 proteins of the IFNγ-primed ASC secretome. Legend: blue, connections for proteins with known interactions based on curated databases; red, connections for proteins with experimentally determined interactions; empty nodes, proteins of unknown 3D structure; filled nodes, known or predicted 3D structure

A selection of 200 inflammatory and growth factors, chemokines, receptors, and cytokines was scored on ASC-conditioned medium. Fifty-seven molecules were found above the ELISA detection threshold at varying levels of intensity in all ASC samples (Additional file 1: Table 1 and Additional file 2). A pattern of similarity more than divergence between samples was observed since a correlation analysis showed a mean *R* value of 0.92 ± 0.06. Consequently, an average intensity value was calculated for each factor in order to provide a guide to its level. In 48 h, per million cells, 4 factors were secreted with an average amount superior to 100 ng, namely FST (531 ± 220), TIMP2 (200 ± 21), IGFBP4 (144 ± 40), and SERPINE1 (101 ± 4). The average amount of 10 factors was between 10 and 100 ng: IGFBP6 (85 ± 3), IL6ST (46 ± 16), TIMP1 (31 ± 3), IL6 (22 ± 28), CTSS (19 ± 9), PLG (17 ± 2), TNFRSF1A (15 ± 6), CCL2 (13 ± 3), DKK1 (10 ± 2), and IGFBP3 (10 ± 4). The average amount of 18 factors was between 1 and 10 ng and others 26 below 1 ng. A functional protein association network analysis (Fig. 2a) of the 57 proteins, regardless their expression amount and based on known interactions given by experimental and database sources, identified a specific cluster (Fig. 2b), composed in its core by CXCL1/5/8/12/16 and CCL4/5/13/27, all involved in chemotaxis and immune response, and, more distant, CTSS/TIMP2, related to extracellular matrix remodeling.

To give a more detailed overview of the secretome potential that goes beyond the association networks, a Gene Ontology (GO) analysis was performed against the background population (200 assayed factors), being aware that a selected protein list was scored. When assessing the 14 most abundant proteins (> 10 ng per million cells), two functional categories resulted significantly enriched: extracellular matrix disassembly (GO:0022617; *p* value 5.5e−04; PLG, IL6, CTSS, and TIMP1/2) and cellular component disassembly (GO:0022411; 6.7e−04; PLG, IL6, CTSS, TIMP1/2, DKK1). These results, in agreement with the association network, suggest a potential in the regulation of processes involved in matrix remodeling. Analyzing the molecules with intermediate expression (1–10 ng), no enriched functional categories were found. To get more insights, biological processes (BP) were scored using the PANTHER GO-Slim Biological Processes tool. The BP encompassing the highest number of proteins, as previously emerged for the association network cluster, were connected with chemotaxis/motility and immune response. Specifically, granulocyte (GO:0071621; CCL4/5 and CXCL1/5/8) and leukocyte (GO:0030595; CCL4/5, CXCL1/5/8, and VEGFA) chemotaxis, cell migration (GO:0016477) and cell motility (GO:0048870), both defined by CCL4/5, CXCL1/5/8, VEGFA, AXL, and FLT1. For the inflammatory response (GO:0006954), again, CCL4/5 and CXCL1/5/8 defined this category. These results suggest a potential effect of ASC-secreted factors on host immune cells. Finally, the functional categories related to the less abundant (< 1 ng) ones were analyzed. No enriched GO terms were obtained. Mining the BP containing the highest number of members, immune system categories arose: response to stimulus (GO:0050896; CCL3/13/27, CD14, IL1B, SIGLEC5, TGFB1, and TNFSF14), with some of the factors (CCL3/13, CXCL16, IL1B, and TYRO3) also defining cell migration (GO:0016477) and motility (GO:0048870). Notably, CCL3/13, CXCL16, and IL1B were part of leukocyte chemotaxis (GO:0030595) and migration (GO:0050900). Again, immune cells resulted a target of ASC secreted factors, even when expressed at low levels.

### IFNγ influence on ASC-secreted molecules

**Fig. 3:**
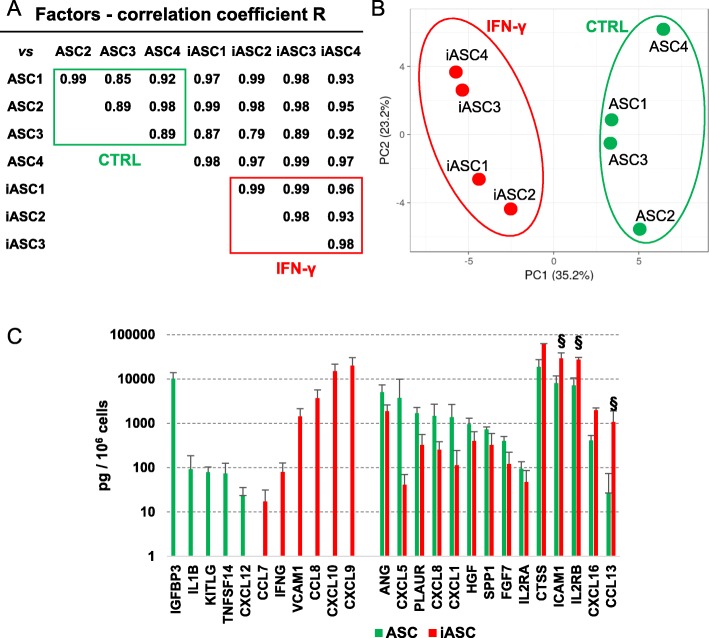
Cytokine and growth factor secretion in ASCs and inflammatory modulation. **a** Correlation of the 57 factors identified by ELISA, in ASCs and iASCs. Absolute values are shown in Additional file 2: Table 2. **b** Principal component analysis demonstrating IFNγ effect on global factor expression. PCA was generated after row centering. **c** Newly secreted, depleted, or modulated factors after IFNγ. All modulated factors have a statistical significance with a *p* value < 0.05 (see Additional file 2: Table 2), with the exception of ICAM1, ILR2RB, and CCL13 (*§ p* value < 0.1)

After IFNγ preconditioning (48 h, 10 ng/ml), 58 factors were scored (Additional file 2: Table 2), with a high correlation between inflamed samples (mean *R* of 0.97 ± 0.02). PCA analysis demonstrated that in a context of overall similarity considering all 8 samples (± inflammation, mean *R* of 0.95 ± 0.06) (Fig. 3a), IFNγ priming allowed for a sharp discrimination of samples (Fig. 3b). Inflammation leads to loss of few factors (Fig. 3c): IGFBP3, IL1B, KITLG, TNFSF14, and CXCL12. On contrary, CXCL10 (14.9 ± 6.6 ng) and CXCL9 (19.9 ± 10.4 ng) appeared at high levels and VCAM1 (1.4 ± 0.7 ng) and CCL8 (3.7 ± 1.9 ng) at moderate amounts, whereas IFNG and CCL7 were barely detectable. Regarding modulated factors (Fig. 3c), in the untreated samples > 10 ng group, only CTSS significantly (*p* value < 0.05) increased (3.71-fold). ICAM1 and IL2RB increased in all samples, 4.22- and 4.64-fold, respectively, reaching high levels (> 10 ng) although without statistical significance (*p* value between 0.1 and 0.05). Conversely, few factors reduced their expression: ANG, CXCL5, PLAUR, CXCL8, and CXCL1. For the low expressed factors in unprimed ASCs (< 1 ng), CXCL16 (5.28-fold; 2.0 ± 0.2 ng) and CCL13 (183.81-fold; 1.1 ± 0.8 ng) increased their expression, although the last one have a *p* value of 0.0808. Other low abundant factors significantly reduced their expression, remaining barely detected molecules. The functional protein association network analysis resulted very similar to the one previously obtained (Fig. 2c), with the identification of a tight cluster (Fig. 2d) composed of CXCL1/5/8/9/10/16 and CCL4/5/13/27, and more distant, CTSS/TIMP2, again emphasizing IFNγ-primed secretome influence on chemotaxis/immune response and matrix remodeling.

A more detailed Gene Ontology enrichment analysis on the newly synthesized molecules did not find over- or under-represented terms. Notably, PANTHER was able to identify BP encompassing the majority of factors, as response to stimulus (GO:0050896), granulocyte (GO:0071621) and leukocyte (GO:0050900) chemotaxis, cell migration (GO:0016477) and motility (GO:0048870), and inflammatory response (GO:0006954), all defined by (CCL7/8 and CXCL9/10). Therefore, staying in the rut of previously observed secretome, IFNγ may further increase modulation on the immune system cells. Regarding the factors that are lost with inflammation, for both GO enrichment analysis and BP scoring, no significant terms were found.

### Characterization of ASC-derived extracellular vesicles

**Fig. 4:**
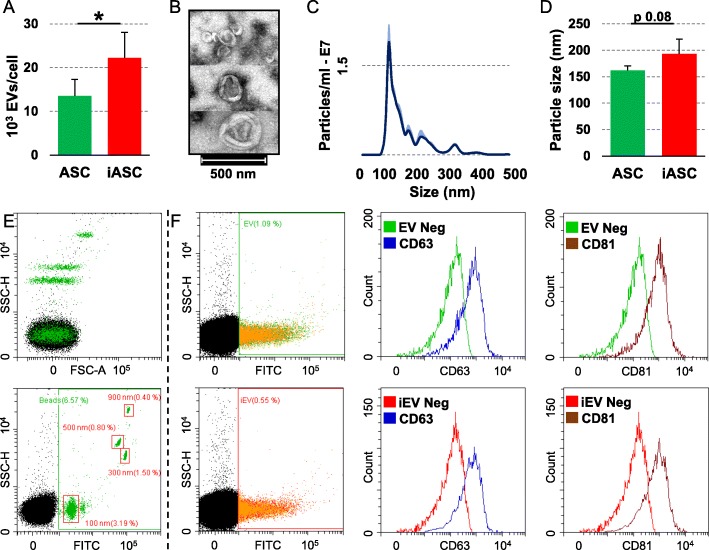
ASC-EV characterization. **a** Number of EVs secreted per cell in 48 h. **b** Transmission electron micrographs of ASC-derived vesicles showing particles with characteristic cup-shaped morphology. **c** Size distribution of nanoparticles by NanoSight particle tracking analysis. **d** Mean particle size analysis from NTA data. **e** Setting up the EV-dedicated flow cytometer. The resolution of the reference bead mix indicates the flow cytometer performance in light scattering at default settings. The top cytogram shows the side scatter height (SSC-H) versus forward scatter area (FSC-A). The bottom cytogram depicts the SSC-H versus 535/35 (green fluorescence triggering) channel. Four fluorescent populations (100, 300, 500, and 900 nm) were resolved from the instrument noise. **f** Flow cytometry analysis of ASC-EVs and iASC-EVs. EVs were stained with CFSE to allow identification and gating of vesicles in the FITC channel. After gating, CFSE+ EVs showed positive extracellular vesicle defining molecules CD63 and CD81. Representative cytograms are presented

ASCs release around 13,500 extracellular vesicles (from now on termed EVs) per cell in 48 h. IFNγ preconditioning significantly (*p* value < 0.05) increases vesicle (iEV) production of a 1.7 ± 0.3 ratio, reaching 22,200 particles/cell (Fig. 4a). Isolated EVs were analyzed by transmission electron microscopy and nanoparticle tracking analysis (NTA). EVs exhibited the characteristic cup-shape morphology (Fig. 4b) and were within the reported size range (50–400 nm in diameter), with enrichment in the small ones (< 150 nm) (Fig. 4c). After inflammation, iEVs resulted larger (193 ± 28 vs 162 ± 9 nm), although without reaching statistical significance (*p* value of 0.0796) (Fig. 4d). After calibration of the flow cytometer to detect particles in the nanometer range (100 to 900 nm, Fig. 4e), both EVs and iEVs demonstrated to express vesicle markers CD63 (53.1% ± 0.3 for EVs and 51.1% ± 0.8 for iEVs) and CD81 (61.5% ± 1.5 for EVs and 60.4% ± 1.1 for iEVs) at comparable levels (Fig. 4f), consistent with previously reported characteristics of extracellular vesicles.

### EV-associated miRNAs

**Fig. 5:**
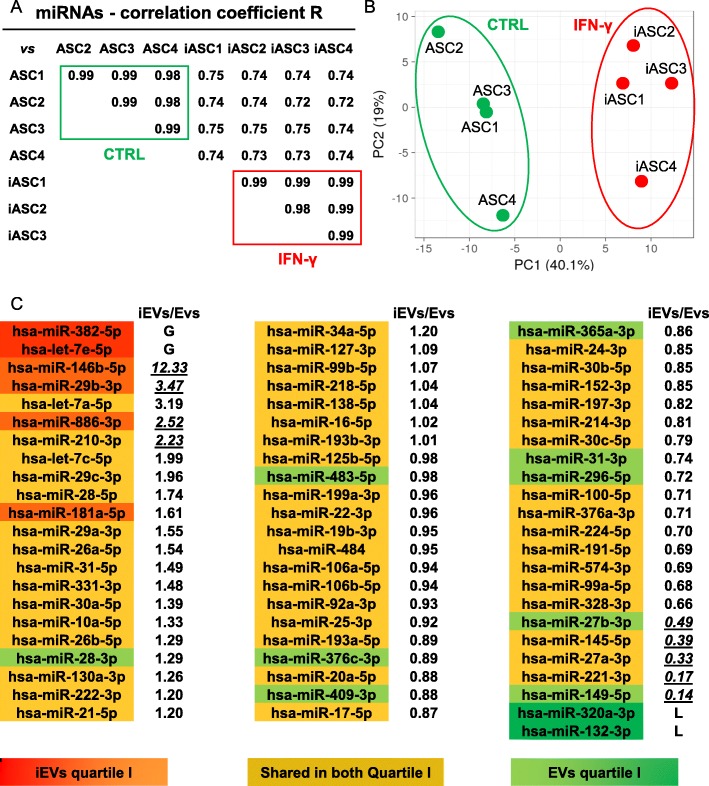
Influence of inflammation on iASC-EVs miRNA profile. **a**, **b** Correlation and principal component analysis of the C_RT_ values of miRNAs after global mean normalization. For PCA, values for each miRNA were centered. miRNAs are shown in Additional file 3: Table 3. **c** Differential expression or gain (G)/loss (L) for miRNAs in the first quartile of expression after IFNγ. Underlined values are those with statistically significant *p* value < 0.05 (Additional file 3: Table 3)

A total of 242 miRNAs for EVs and 222 for iEVs were detected (Additional file 3: Table 3). Inter-correlation analysis showed high conservation (*R* of 0.99 ± 0.01) for EVs and iEVs groups (Fig. 5a). After IFNγ stimulation, both correlation (*R* of 0.74 ± 0.01) and PCA showed distinct clusters (Fig. 5a, b), due to 10 miRNAs appearing and 30 disappearing and 7 candidates significantly (*p* value < 0.05, fold > 2) overexpressed (miR-146b-5p/146b-3p/155-5p/210-3p/29b/3p/455-5p/886/3p) and 9 reduced (fold < 2) (miR-145-5p/149-5p/199a-5p/221-3p/27a-3p/27b-3p/345-5p/503-5p/671-3p), after inflammation.

Next, we scored embedded miRNAs significance. Recent findings suggested that even for most abundant EV-conveyed miRNAs, there is around 1.3 molecules per MSC vesicle [39], and 100 EVs would be needed to transfer one copy of an abundant miRNA [40]. Due to these premises, only highly expressed molecules, such as those laying in the first quartile of expression (61 and 56 miRNAs for EVs and iEVs, respectively, covering 96.8% and 95.9% of the genetic message), were considered (Fig. 5c). Few miRNas were not included in both lists, although significant difference in the expression or loss/gain was a rare event. miR-320a-3p and miR-132-3p were not expressed at all in iEVs, whereas miR-27b-3p and miR-149-5p resulted significantly (ratio < 0.5, *p* value < 0.05) downregulated (ratio of 0.49 and 0.14, respectively) under inflammation. Conversely, iEVS-enriched miR-382-5p and let-7e-5p were not amplified at all in unprimed EVs, with miR-146b-5p/29b-3p/886-3p less expressed (0.08, 0.29, and 0.40) without inflammation. Due to these small differences, miRNAs from both quartiles were considered together, defining a group of 67 molecules (Fig. 5c). In this list, 5 miRNAs resulted downregulated (miR-221-3p/145-5p/27a-3p/27b-3p/149-5p) and 4 upregulated (miR-210-5p/146b-5p/29b-3p/886-3p) by IFNγ, with miR-320a-3p and miR-132-3p EVs specific and miR-382-5p and let-7e-5p iEVs distinctive.

### EV-embedded miRNAs target analysis and IFNγ influence

Sixty-three miRNAs obtained from combined first quartiles candidates, excluding those unique to EVs (miR-320a-3p/132-3p) or iEVs (has-miR-382-5p/let-7e-5p), were analyzed with mirPath to identify potential BP targets based on in silico predicted miRNA-mRNA interactions (Additional file 4: Table 4). Out of 24 enriched (*p* value < e−3) BP, the top five GO terms were cellular nitrogen compounds metabolic process (GO:0034641; 1.8e−157; 2484 genes), biosynthetic process (GO:0009058; 6.9e−109; 2088 genes), cellular protein modification process (GO:0006464; 3.1e−95; 1304 genes), small molecule metabolic process (GO:0044281; 1.6e−58; 1187 genes), and symbiosis (GO:0044403; 7.3e−43; 299 genes). Although with lower significance, three terms were related with those identified for secreted factors: immune system process (GO:0002376; 5.4e−12; 717 genes), cell motility (GO:0048870; 3.8e−10; 275 genes), and extracellular matrix organization (GO:0030198; 2.3e−4; 162 genes). miR-320a-3p/132-3p and miR-382-5p/let-7e-5p resulted able to potentially regulate 1006/703 and 28/1692 genes, respectively, allowing the definition of a relevant number of significant BP (18 and 24, respectively). This lead to 16 GO terms shared with the previous analysis, with 13 in the top of the ranking for both EVs and iEVs. Notably, immune system process was again predicted to be significantly regulated with a higher number of genes for iEVs (156 vs 137), whereas extracellular matrix organization was identified only for iEVs list, with low *p* value (2.3e−3; 40 genes). Nevertheless, even with a reduced number of miRNAs, as 2 in our case, the most significantly enriched GO terms are similar for both category and heterogeneity, making almost impossible to predict either subtle or even general differences in potency without the definition of a specific target disease/tissue or transcript list.

Due to the above findings, we focused our attention on miRNA-mRNA regulation in OA setting, being matrix-enriched cartilage and immune cells among the main pathological players and the majority of authorized MSC-based products related to musculoskeletal indications. Ingenuity Pathway Analysis on experimentally verified miRNA-mRNA interactions identified 42 miRNAs related to “skeletal and musculoskeletal disorders.” Of these miRNAs, 31 were directly or indirectly involved in the “osteoarthritis pathway” (Additional file 5: Table 5). A more refined search for miRNAs linked to “inhibition of matrix metalloproteases” showed subgroups defined by 8 miRNAs. With respect to “inflammatory response,” 41 miRNAs directly regulate mRNAs covering different pathways: “inflammasome” was represented by 4 miRNAs, “chemokine signaling” 12 miRNAs, “altered T and B cell signaling” 9 miRNAs, and finally, “role of macrophages, fibroblasts, and endothelial cells” 31 miRNAs (Additional file 5: Table 5). Therefore, again, extracellular matrix and immune system resulted preferential targets.

To get further insights on the specifically regulated biological processes, a more refined and literature-based analysis was performed on miRNAs reported to directly regulate specific tissues or cell types [41, 42]. Ten and 7 miRNAs were associated with cartilage protection or degenerescence, respectively (Table 1). For protective molecules, IFNγ reduced the expression of miR-149, involved in inflammation, and increased the amount of miR-210, also involved in inflammation and apoptotic processes. Concerning destructive functions, IFNγ reduces miR-145 that is involved in chondrocyte differentiation. As a whole message, protective miRNAs represented the 34.71% of EVs genetic weight vs the 13.28% of the destructive ones, indicating a strong preponderance of the salvage functions. Further, preconditioning slightly increases the percentage of miRNAs in both categories (36.41% vs 14.83%), maintaining the delta between protection and degeneration at similar levels. Regarding immune system, we investigated miRNA-regulating factors orchestrating the macrophage M1 (pro-inflammatory) to M2 (anti-inflammatory) switch and phenotype (Table 2), being the imbalance a crucial factor linked to severity level of knee osteoarthritis [43]. Seven M1-related miRNAs were found in ASC-EVs, with IFNγ reducing the levels of M1-promoting miR-27a-3p/miR-27b-3p and miR-145-5p and increasing miR-29b-3p. For M2 miRNAs, 5 candidates were identified in the vesicles, having miR-146b-5p augmented after inflammatory priming. As a whole, in EVs M2-related miRNAs, weight accounted for 26.22% vs 4.49% for M1 molecules, indicating a clear preponderance for resolving mechanisms. IFNγ was able to improve the trend, being M2 vs M1 delta increased (28.76% vs 3.61%). Therefore, overall in the OA settings, EV-miRNAs may have protective roles on both cartilage and inflammatory macrophages, with preconditioning able to tip the trend towards an even higher protective phenotype.

**Table 1:** EV-miRNAs in the first quartile of expression that are involved in cartilage-protective/degenerative mechanisms

	% weight EVs/iEVs	Down IFNγ	Up IFNγ	Only EVs	Only iEVs	Target genes	Functions
Cartilage protective							
hsa-miR-21-5p	5.13/6.73					*GAS5*, *GDF5*	Autophagy
hsa-miR-222-3p	5.74/7.61					*MMP13*, *HDAC4*	Cartilage degradation
hsa-miR-138-5p	0.19/0.21					*SP1*, *HIF-2Α*	Chondrocyte differentiation
hsa-miR-24-3p	19.63/18.47					*P16INK4A*	Chondrocyte differentiation and apoptosis
hsa-miR-210-3p	0.23/0.56		X			*DR6*, *NF-ΚB*	Inflammation, chondrocyte apoptosis
hsa-miR-26a-5p	0.64/1.06					*NF-ΚB*, *CD200*, *COL10A1*, *COL9A1*, *CTGF*	Inflammation, modulate ECM homeostasis
hsa-miR-130a-3p	0.57/0.78					*TNF-Α*	Inflammation
hsa-miR-149-5p	0.13/0.02	X				*TNFΑ*, *IL-1*, *IL-6*	Inflammation
hsa-miR-199a-3p	0.92/0.97					*COX-2*	Inflammation
hsa-miR-320-3p	0.00/0.53			X		*MMP13*	Matrix degradation
Cartilage destructive							
hsa-miR-21-5p	5.13/6.73					*GDF5*	Chondrocyte differentiation and homeostasis
hsa-miR-145-5p	1.84/0.79	X				*SOX9*, *SMAD3*	Chondrocyte differentiation and homeostasis
hsa-miR-16-5p	0.40/0.44					*SMAD3*	Chondrocyte differentiation and homeostasis
hsa-miR-193b-5p	5.00/5.56					*TGF-Β2*, *TGF-ΒR3*, *SOX9*, *COL2*	Chondrocyte differentiation and homeostasis
hsa-miR-29b-3p	0.07/0.27					*SMAD*, *NF-ΚB*, *WNT*	Chondrocyte differentiation and homeostasis
hsa-miR-34a-5p	0.70/0.93					*COL2Α1*, *INOS*	Chondrocyte apoptosis
hsa-miR-483-5p	0.12/0.11					*BMP7*, *TGFΒ*, *IL-1Β*, *MMP13*	Inflammation

**Table 2:** EV-miRNAs in the first quartile of expression that are involved in macrophage M1 or M2 phenotype

	% weight EVs/iEVs	Down IFNγ	Up IFNγ	Only EVs	Only iEVs	M1 promoting	M1 suppressing	M2 promoting	M2 suppressing
M1 phenotype									
miR-29b-3p	0.07/0.27		X			X			
miR-145-5p	1.84/0.79	X				X			
miR-27a-3p	0.90/0.33	X				X			X
miR-27b-3p	0.32/0.18	X				X			X
miR-130a-3p	0.57/0.78					X			X
miR-26a-5p	0.64/1.06								X
miR-26b-5p	0.15/0.20								X
M2 phenotype									
miR-24-3p	19.63/18.47						X	X	
miR-146b-5p	0.04/0.51		X				X	X	
miR-181a-5p	0.11/0.18						X	X	
miR-34a-5p	0.70/0.93							X	
miR-222-3p	5.74/7.61							X	

### Validation of secretome effects and IFNγ priming on inflamed macrophages

**Fig. 6:**
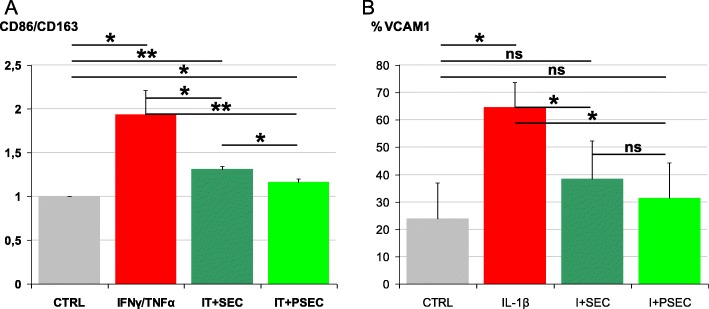
Secretome effects on inflamed macrophages and chondrocytes. **a** Macrophages (CTRL) were treated for 24 h with inflammatory cytokines without (IFNγ/TNFα) and with secretome (IT+SEC) or IFNγ-primed secretome (IT+PSEC). Untreated cells were used as control (CTRL). CD86 (M1 phenotype) and CD163 (M2 phenotype) were detected by flow cytometry. Values on the *y*-axis are intended as ratios obtained comparing CD86 and CD163 median fluorescence intensities subtracted of their unstained control values and arbitrarily set as 1 for CTRL. Increased CD86/CD163 ratio is an indication of M1 phenotype polarization. **b** Chondrocytes (CTRL) were treated with inflammatory cytokine without (IL-1β) and with secretome (I+SEC) or IFNγ-primed secretome (I+PSEC). VCAM1 was detected by flow cytometry. Values on the *y*-axis show the percentage of VCAM1-positive cells. *N* = 3, *p < 0.05, **p < 0.01; ns, not significant

In silico data suggested an anti-inflammatory capacity for both secreted factors and EV-embedded miRNAs. Moreover, IFNγ priming appeared to potentiate the secretome healing properties. To validate bioinformatics, human macrophages were treated for 24 h with IFNγ and TNFα, and both M1 marker CD86 and M2 marker CD163 levels were tested by flow cytometry. As expected, inflammation resulted in a significant CD86 increase (1.41 ± 0.15 ratio vs unstimulated macrophages, *p* value < 0.05) and CD163 decrement (0.74 ± 0.18, *p* value < 0.1). The CD86/CD163 ratio, with unstimulated macrophages set as 1, significantly augmented to 1.9, a clear indication of a M1 polarization (Fig. 6a). When secretome was added together with inflammatory stimuli, CD163 reduction was completely abolished with respect to unstimulated macrophages (1.07 ± 0.24 ratio, *p* value > 0.1) due to a 44 ± 5% (*p* value < 0.01) increase of the M2 marker vs IFNγ/TNFα cells. Eventually, when IFNγ-primed secretome was tested, CD163 levels further increased (60 ± 11%, *p* value < 0.05) with a significant upregulation with respect to non-primed secretome (11% ± 3%, *p* value < 0.05), meaning an almost complete prevention of the polarization of macrophages to the M1 pro-inflammatory phenotype as also indicated by the CD86/CD163 arbitrary ratio of 1.1 (Fig. 6a).

### Validation of secretome effects and IFNγ priming on inflamed chondrocytes

Secretome activity was also monitored on inflamed chondrocytes. A chronic and low level IL-1β (25 pg/ml) treatment was used accordingly to a protocol developed in our laboratory to mimic the osteoarthritis environment on synoviocytes [27]. VCAM1 positivity was tested by flow cytometry due to its responsiveness to chondrocyte inflammation and response to environmental metabolic alterations [44, 45]. One week of IL-1β treatment was able to significantly (*p* value < 0.05) modulate VCAM1 expression increasing the percentage of positive cells from 24 ± 13% to 65 ± 9% (Fig. 6b). When the secretome was added, after 4 days, even in presence of the inflammatory stimulus, the number of VCAM1+ chondrocytes reduced to 38 ± 14%. The secretome + IL-1β vs IL-1β ratio of VCAM1+ cells was 0.58 ± 0.15 (*p* value < 0.05). Finally, IFNγ-primed secretome further decreased VCAM1+ cells (31 ± 13%, *p* value < 0.05) and the ratio vs IL-1β cells (0.47 ± 0.14, *p* value < 0.05). Of note, although not significant when comparing gross VCAM1+ percentages, the IFNγ-primed secretome vs secretome ratio resulted 0.80 ± 0.05 (*p* value < 0.05), suggesting a more potent anti-inflammatory activity after priming and confirming in silico data.

## Discussion

In this work, both soluble factors and EV-shuttled miRNAs have been characterized in serum-free ASC secretome, and the influence of IFNγ preconditioning evaluated. In a context of shared overall protective signals, inflammatory priming was able to tip the balance towards a more pronounced tissue regenerative and anti-inflammatory phenotype. Further, on a general perspective, identified molecules may be a useful array for future in vitro and in vivo potency assays able to define a new generation of secretome-based products and disease-driven clinical targets.

Few factors are secreted at high rates (> 100 ng) and may shuttle a message that goes beyond or drive the global scenario. Follistatin [46] is by far the most abundant (> 500 ng). In an OA mouse model, follistatin reduced synovial inflammation [47] by binding the synovia-derived and macrophage activator activin A [48]. Therefore, follistatin may contribute to observed anti-inflammatory activity of secretome in OA [49]. TIMP2 (200 ng) and TIMP1 (> 30 ng), inhibitors of matrix metalloproteinases (MMPs), may contribute to observed prevention of cartilage degradation in both an in vivo and a phase I/II study [50, 51]. The same paradigm may be also applied for other pathologies involving TIMP/MMP balance as skeletal dysplasias, coronary artery and heart disease, cancer, and brain disorders [52]. IGFBP4 (144 ng), with IGFBP6 (85 ng) and 3 (10 ng), was shown to balance the IGF-dependent induction of CD4+FOXP3+ Tregs given by MSC-conditioned medium in arthritis [53]. The IGF/IGFBP axis may further contribute to properly regulate cartilage and bone homeostasis in OA pathogenesis, where IGF levels are increased [54]. Eventually, Serpin E1 (100 ng) prevents both the formation of plasmin [55] and the activity of MMPs [56]. Consistently, in OA-affected cartilage, Serpin E1 was upregulated and associated with disease severity, balancing matrix deposition/degradation [57]. Moreover, in macrophages, Serpin E1 controls invasion, adhesion, and again remodeling of the extracellular matrix, therefore being a regulator of the inflammatory process [58]. Notably, IFNγ preconditioning neither significantly changed the amount of the most enriched factors nor allowed an already or newly secreted molecule to exceed the 100 ng burden.

Rarely, single molecules and their interactions are able to explain the functional relationships when specific and focused pathways representing spatial and temporal sets of disease-dependent interactions are not scored [59]. Analyzing the data under this system view paradigm, abundant factors (> 10 ng) framed “cellular” and “matrix disassembly” GO terms. This confirms the high overall potency for the secretome in those processes or diseases where active ECM remodeling is the crucial player [60]. Plasminogen [61] was found 5 times more abundant in its active form in OA cartilage, due to both an increase in plasminogen activator urokinase (uPA) and a reduction of inhibitor of plasminogen activator (Serpin E1) levels [62]. Cathepsins degrade extracellular ECM proteins [63] as those in the cartilage [64]. Overall, despite a crucial role in cartilage homeostasis, plasminogen/cathepsin S most studied contribution is related to angiogenesis [65, 66], a process timely and spatially correlated with leukocyte motility [67]. Notably, ASC secretome was demonstrated to show angiogenic potential in vivo and in vitro [68] and chemo-attraction for most of the immune cells [25]. These evidences were here supported by the presence of angiogenic molecules like interleukin-6 and CCL2, in the > 10-ng frame, and angiogenin and VEGF α in the 1- to 10-ng group [69]. Moreover, in the last group, enriched GO categories like “leukocyte” and “granulocyte chemotaxis” and, more in general, “cell migration” and “motility” have been found as highly represented. Interestingly, few factors were shared between these annotations, like CCL4/5 and CXCL1/5/8, all defining the “inflammatory response” group. Supporting this capacity, also CCL2 was highly expressed (13 ng), and in the < 1-ng molecules, “cell motility” and “migration” GO annotations were found, with CCL3/13 and CXCL16 in the “leukocyte chemotaxis” and “migrations” terms. CCL2/13 have their main function in monocyte trafficking, CCL3/4/5 in macrophage and NK cell migration, and CXCL1/5/8 in monocyte and neutrophil trafficking [70]. These data support the chemo-attractive capacity of MSC-conditioned medium that was demonstrated to enhance monocyte/macrophage motility and differentiation [71]. This may be further due to the presence of CSF1, shown to promote the differentiation and survival of monocytes/macrophages [72], and stimulate the anti-inflammatory M2 macrophage polarization supporting a CSF1-mediated reparative/homeostatic state [73]. Intriguingly, a MSC-based engineered cartilage suppressed in vivo inflammation through the alteration of macrophage phenotype and a M1 to M2 transition [74]. Overall, these results again support the immunomodulatory potential of MSC secretome, being aware that a selection of 200 molecules has been assayed and additional cytokine-dependent pathways may integrate the overall message.

Analysis of differentially expressed candidates between IFNγ-activated and naïve MSCs confirmed the up- or downregulation of various immunomodulatory factors, respectively. In the group of highly expressed proteins, only IGFB3 was lost whereas CXCL9/10 de novo appeared at > 10 ng and CCL8 at > 1 ng. Scoring the significance of these induced factors, again “response to stimulus” and “chemotaxis/migration/motility” GO terms emerged, defined by CCL7/8 and CXCL9/10. Interestingly, CXCL9 and 10 are involved T cell recruitment and CCL8 (also called monocyte chemoattractant protein 2) specifically regulates Th2 response [70]. The presence of these factors is in agreement with higher capacity of IFNγ-inflamed MSCs to recruit T cells at their proximity and ability to reduce the symptoms of graft-versus-host disease (GVHD) in a mouse model [75], being CCL8 and CXCL9/10 upregulated in the mouse system after inflammation [76]. T cell attraction and recovery were shown to be coupled with IFNγ-dependent activation of indoleamine 2,3-dioxygenase (IDO) [76], as also observed in our setting. In fact, IDO has been demonstrated to inhibit the proliferation of PBMNC, particularly activated T lymphocytes, and prevent conversion of immunosuppressive Tregs in inflammatory Th1/Th17 cells [11]. Looking at the factors already identified without priming, chemokines involved in neutrophil activity (CXCL1/5/8) showed a significant reduction (still detectable but at < 1 ng) whereas CXCL16 overcame 1-ng burden (5-fold increase). To our knowledge, this is the first report showing an increase for CXCL16. Moreover, with respect to other published datasets dissecting inflamed MSC potency (reviewed in [23]), we observed some discrepancies. In particular, in our experimental conditions, out of reported upregulated molecules, CXCL9/10 confirmed the trend, CCL2/5 were not modulated, CXCL1/5/8/12 behaved in an opposite fashion and reduced, and inflammation related IL6 and IL23A did not change their amount, whereas increase of adhesion molecules (VCAM1 and ICAM1) corroborated the published data. These variations may be due to several factors. First, different inflammatory stimuli may induce alternative responses. As an example, many studies have demonstrated the divergent effects of MSCs priming with pro-inflammatory cytokines as IFNγ, TNF-α, or IL-1β (reviewed in [24]). Second, MSC from distinct sources might respond differently to preconditioning with pro-inflammatory factors [77]. Eventually, many of the works aimed at dissecting secreted factors and cellular response are designed in view of directly utilizing MSCs as cell-based therapy. Therefore, often cytokine and chemokine modulation is studied in the presence of both FBS and the inflammatory insult. In the herein presented approach, that was realized in view of potential therapeutic application of the secretome, both serum and IFNγ had to be removed before conditioned medium collection, thus allowing for a slightly different release/modulation of factors.

Together with secreted factors, MSC-derived EVs have been shown to replicate the therapeutic effects observed with the entire secretome [78] by transfer of DNA, proteins/peptides, lipids, organelles, mRNAs, and miRNAs [79]. In the view of placing EV-miRNA significance in the whole secretome capacity, some pitfalls have still to be overcome. First, there is a lack of consensus regarding miRNA signature among MSC-EVs from different sources [9, 80, 81] or independent labs [9, 80]. Therefore, the delivery of a list of identity marker molecules is still far to be defined. Second, the number of the delivered miRNAs per recipient cell may also impact the selection of important molecules. Recently, it was argued that in MSC-EVs, there is around 1.3 miRNA, even for the most abundant ones [82], and that, on average, 100 EVs would be needed to transfer one copy of a given abundant miRNA [40]. Since the number of incorporated MSC-EVs per cell ranges from few to hundred thousand [8, 27], at least in stromal tissues, this opens the question on the real number of transferred molecules and their biological relevance. For these reasons, the most abundant EV-miRNAs may be those really shuttling a profound biological message. In this view, Baglio and colleagues showed that the 5 most enriched miRNAs accounted for around 50% of the total miRNA reads, whereas in Fang and Ferguson’s datasets, the top 8 and 23 miRNAs represented 40% and 80% of the total, respectively [9, 80, 81]. Here, the first quartile of detected EV-miRNAs accounted for > 95% of the genetic fingerprint.

The effects of MSC-EVs are dependent on both the profiles of their miRNAs and the specific mRNA signature of target cells. Without these premises, herein detected miRNAs could be related to thousands of genes and dozens of GO categories (Additional file 4: Table 4). We therefore addressed OA as a focused condition. Notably, IPA analysis showed that a large number of miRNAs in the first quartile are connected with matrix or immune cell-related pathways and cascades (Additional file 5: Table 5). Nevertheless, a net effect for each single miRNA is barely predictable, and system view including target tissues or cell types is again mandatory. As a whole, EV-miRNas resulted to have a pronounced cartilage-protective features with a prevalence in reducing matrix/cartilage/ECM degradation and cartilage inflammation, together with a very mild detrimental effect on chondrocyte differentiation/homeostasis pathways. In this view, in a meta-analysis studying MSC treatment [83], the improvement in cartilage tissue quality and volume may be, at least in part, ascribed to the combined action of matrix-protective factors and miRNAs affecting chondrocyte homeostasis. Regarding macrophage polarization, abundant EVs have a prevalence for M2-protective signals that are increased by IFNγ preconditioning, a situation similar to what may happen when MSCs are injected in the diseased and inflamed joint cavity. In this perspective, in the synovial fluid of an OA porcine model, MSC-EVs were able to mobilize monocytes [84] that, in view of herein presented results, could polarize to an anti-inflammatory M2 phenotype. This was confirmed in an in vivo model of induced OA where amniotic fluid-derived MSC-EVs allowed macrophage M2 polarization with an almost complete restoration of cartilage, enhancing pain tolerance [85]. Thus, the combination of both M2 macrophage polarizing and cartilage protecting miRNAs and cytokines may at least in part explain the result of the Iijima meta-analysis on pain, where a significant improvement has been reported [83].

Overall, the combination of secreted factors and EV-miRNAs, and therefore the ASC secretome as a whole, in silico appeared to have both protective and anti-inflammatory activities on macrophages and chondrocytes, suggesting its use as therapeutic product for joint diseases, as well as the inflammatory priming as an effective strategy to improve its efficacy. These bioinformatics results and those in vitro for chondrocytes and macrophages are in agreement with and integrate recent reports on the efficacy of MSC secretome, and its priming, for joint diseases which was shown to inhibit both chondrocyte catabolic and inflammatory markers and macrophage activation [49, 71, 86, 87]. Likewise, in other disease settings, several priming approaches such as lipopolysaccharides, IL-1β, or IFNγ+TNFα were able to better induce M2 phenotype and IL-10 secretion [88–91]. Altogether, these evidences confirm both anti-inflammatory ASC secretome potential and the enhancing ability of IFNγ priming.

## Conclusions

There is still a wide knowledge gap between a general definition of MSC-derived secretome efficacy and its modulation after priming to explain or even predict induced alteration of pathological pathways in the target cells when used as a medicinal product. Herein, presented results clearly showed how a deep characterization of both the clinical products and the diseased cells or tissue types is crucial to envision the therapeutic efficacy, as we observed for OA-affected cartilage and macrophages. Further studies will be needed to describe in more detail the secretome at proteic, lipidic, or nucleic acid levels in order to couple the overall fingerprint with the wide array of pathologies potentially treatable with off-the-shelf MSC cell-free products.

## Supplementary information

**Additional file 1: Table 1**. List of detected soluble factors and their molecular function in ASC/iASC secretome.

**Additional file 2: Table 2**. Expression values and ratios for detected soluble factors in ASC/iASC secretome.

**Additional file 3: Table 3**. Expression values and ratios for miRNAs in ASC/iASC EVs.

**Additional file 4: Table 4**. Potential BP targets based on in silico predicted miRNA-mRNA interactions.

**Additional file 5: Table 5.** Ingenuity Pathway Analysis on experimentally verified miRNA-mRNA interactions.

## Data Availability

The datasets and raw data generated and/or analyzed during the current study are available in the OSF repository, https://osf.io/2sg9e/

## Abbreviations

MSC: Mesenchymal stromal cells
ASC: Adipose-derived MSC
BMMSC: Bone marrow-derived MSC
EV: Extracellular vesicles
OA: Osteoarthritis
GO: Gene Ontology
MMP: Matrix metalloproteinase
TIMP: Tissue inhibitor of metalloproteinase
