# Supplementary material for: Inflammatory priming enhances mesenchymal stromal cell secretome potential as a clinical product for regenerative medicine approaches through secreted factors and EV-miRNAs: the example of joint disease
Source: Stem Cell Res Ther. 2020 Apr 28;11:165. doi: 10.1186/s13287-020-01677-9 (PMC7189600; doi:10.1186/s13287-020-01677-9)
Supplement: Supplementary file 1 — Additional file 1: Table 1. List of detected soluble factors and their molecular function in ASC/iASC secretome. [file 13287_2020_1677_MOESM1_ESM.docx]

| FACTOR | FUNCTION |
| --- | --- |
| FST | Follistatin. Neutralize Activins and modulates transforming growth factor (TGF)-β superfamily signaling. |
| TIMP2 | Metalloproteinase inhibitor 2. Inhibitor of matrix metalloproteinase (MMP) involved in degradation of the extracellular matrix. |
| IGFBP4 | Insulin-like growth factor-binding protein 4. Bind both insulin-like growth factors (IGFs) I and II. |
| SERPINE1 | Plasminogen activator inhibitor 1. Serine proteinase inhibitor of tissue plasminogen activator (tPA) and urokinase (uPA). |
| IGFBP6 | Insulin-like growth factor-binding protein 6. Bind both insulin-like growth factors (IGFs) I and II. |
| IL6ST | Interleukin-6 receptor subunit beta. Part of the cytokine receptor complex. |
| TIMP1 | Metalloproteinase inhibitor 1. Matrix metalloproteinases (MMP), peptidase involved in degradation of the extracellular matrix. |
| IL6 | Interleukin-6. Interleukin that acts as both a pro-inflammatory cytokine and an anti-inflammatory myokine. |
| CTSS | Cathepsin S. Lysosomal cysteine protease that participates in the degradation of antigenic proteins for presentation to the MHC class II. |
| PLG | Plasminogen. Inactive precursor of plasmin, a potent serine protease involved in the dissolution of fibrin blood clots. |
| TNFRSF1A | Tumor necrosis factor receptor superfamily member 1A. TNF receptor superfamily of proteins. |
| CCL2 | C-C motif chemokine 2. Also referred as MCP1, recruits monocytes, memory T cells, and dendritic cells to the sites of inflammation. |
| DKK1 | Dickkopf-related protein 1. Antagonize the Wnt/β-catenin pathway via a reduction in β-catenin and an increase in OCT4 expression. |
| IGFBP3 | Insulin-like growth factor-binding protein 3. Bind both insulin-like growth factors (IGFs) I and II. |
| ICAM1 | Intercellular adhesion molecule 1. Soluble form acts as a counter-receptor for the lymphocyte function-associated antigen (LFA-1). |
| IL2RB | Interleukin-2 receptor subunit beta. Involved in T cell-mediated immune responses |
| FLT1 | Vascular endothelial growth factor receptor 1. Plays an important role in angiogenesis and vasculogenesis, |
| ANG | Angiogenin. Potent stimulator of new blood vessels through the process of angiogenesis. |
| GDF15 | Growth/differentiation factor 15. Belongs to the transforming growth factor beta superfamily. Involved in inflammation and apoptosis. |
| OPG | Tumor necrosis factor receptor superfamily member 11B. Regulation of bone density immune system development and signalling. |
| IL1RL1 | Interleukin-1 receptor-like 1. Induced by proinflammatory stimuli, and may be involved in the function of helper T cells. |
| CXCL5 | C-X-C motif chemokine 5. Recruit neutrophils, to promote angiogenesis and to remodel connective tissues. |
| CSF1 | Macrophage colony-stimulating factor 1. Controls the production, differentiation, and function of macrophages. |
| SHH | Sonic hedgehog protein. Instrumental in patterning the early embryo. |
| CCL5 | C-C motif chemokine 5. Chemoattractant for blood monocytes, memory T helper cells and eosinophils. |
| PLAUR | Urokinase plasminogen activator surface receptor. Influence plasminogen activation and localized degradation of the extracellular matrix. |
| VEGFA | Vascular endothelial growth factor A. Growth factor that induces proliferation and migration of vascular endothelial cells, angiogenesis. |
| CXCL8 | Interleukin-8 (IL-8). Major mediator of the inflammatory response. Chemotactic factor by guiding the neutrophils to the site of infection. |
| CXCL1 | C-X-C motif chemokine 1. Role in inflammation and as a chemoattractant for neutrophils. |
| TNFRSF1B | Tumor necrosis factor receptor superfamily member 1B. Potentiate TNF-induced apoptosis. |
| AXL | Tyrosine-protein kinase receptor UFO. Involved in growth, migration, aggregation and anti-inflammation. |
| CCL4 | C-C motif chemokine 4. Chemokinetic and inflammatory functions. |
| CD14 | Monocyte differentiation antigen CD14. Mediate the innate immune response. |
| EGFR | Epidermal growth factor receptor. Involved in cell proliferation. |
| TYRO3 | Tyrosine-protein kinase receptor TYRO3. Regulates many physiological processes including cell survival, migration and differentiation. |
| HGF | Hepatocyte growth factor. Regulate cell growth, cell motility and morphogenesis in numerous cell and tissue types. |
| CD40 | Tumor necrosis factor receptor superfamily member 5. T cell-dependent immunoglobulin class switching, memory B cell development. |
| SPP1 | Osteopontin. Upregulates expression of interferon-gamma and interleukin-12. |
| TREM1 | Triggering receptor expressed on myeloid cells 1. Amplifies neutrophil and monocyte-mediated inflammatory responses. |
| LIF | Leukemia inhibitory factor. Induce the terminal differentiation of myeloid leukemic cells. |
| TGFB1 | Human TGF-beta 1. Regulates cell proliferation, differentiation and growth, and can modulate expression of IFNg and TNFa. |
| VCAM1 | Vascular cell adhesion protein 1. Mediates leukocyte-endothelial cell adhesion and signal transduction. |
| MIF | Macrophage migration inhibitory factor. Involved in cell-mediated immunity, immunoregulation, inflammation and macrophage function. |
| CXCL16 | C-X-C motif chemokine 16. Induces a strong chemotactic response. |
| SIGLEC5 | Sialic acid-binding Ig-like lectin 5. Inhibits the activation of several cell types including monocytes, macrophages and neutrophils. |
| FGF7 | Fibroblast growth factor 7. Possess broad mitogenic and cell survival activities. |
| FAS | Tumor necrosis factor receptor superfamily member 6. Play a central role in the physiological regulation of programmed cell death. |
| IL23A | Interleukin-23 subunit alpha. Stimulate the production of interferon-gamma (IFNG) and acts on memory CD4(+) T cells. |
| ENG | Endoglin (CD105). Major glycoprotein of the vascular endothelium. |
| CCL27 | C-C motif chemokine 27. Chemotactic for skin-associated memory T lymphocytes. |
| IL2RA | Interleukin-2 receptor subunit alpha. Present on activated T and B cells, some thymocytes, myeloid precursors, and oligodendrocytes. |
| IL1B | Interleukin-1 beta. Mediator of the inflammatory response, and is involved in cell proliferation, differentiation, and apoptosis. |
| KITLG | Kit ligand. Involved in cell migration. |
| TNFSF14 | Tumor necrosis factor ligand superfamily member 14. Activation of lymphoid cells and stimulate the proliferation of T cells. |
| CCL3 | C-C motif chemokine 3. Plays a role in inflammatory responses through binding to the receptors CCR1. |
| CCL13 | C-C motif chemokine 13. Chemotactic activity for monocytes, lymphocytes, basophils and eosinophils, but not neutrophils. |
| CXCL12 | Stromal cell-derived factor 1. Stimulates migration of monocytes and T-lymphocytes through its receptors, CXCR4 and ACKR3. |
| IL15 | Interleukin-15. Regulates T and natural killer cell activation and proliferation. |
| IFNG | Interferon gamma. Potent activator of macrophages. |
| CXCL10 | C-X-C motif chemokine 10. Stimulation of monocytes, natural killer and T-cell migration, and modulation of adhesion molecule expression |
| CCL7 | C-C motif chemokine 7. Attracts macrophages during inflammation and substrate of matrix metalloproteinase 2. |
| CXCL9 | C-X-C motif chemokine 9. Chemoattractant for lymphocytes but not for neutrophils. |
| CCL8 | C-C motif chemokine 8. Chemotactic activity for monocytes, lymphocytes, basophils and eosinophils. |
