# Supplementary material for: Inflammatory priming enhances mesenchymal stromal cell secretome potential as a clinical product for regenerative medicine approaches through secreted factors and EV-miRNAs: the example of joint disease
Source: Stem Cell Res Ther. 2020 Apr 28;11:165. doi: 10.1186/s13287-020-01677-9 (PMC7189600; doi:10.1186/s13287-020-01677-9)
Supplement: Supplementary file 2 — Additional file 2: Table 2. Expression values and ratios for detected soluble factors in ASC/iASC secretome. [file 13287_2020_1677_MOESM2_ESM.docx]

|  | **ng per 10^6^ cells in 48 hours** | | | | | | | |  |  |  |
| --- | --- | --- | --- | --- | --- | --- | --- | --- | --- | --- | --- |
| **FACTOR** | **ASC1** | **ASC2** | **ASC3** | **ASC4** | **iASC1** | **iASC2** | **iASC3** | **iASC4** | **iASC/ASC** | **SD** | **p-value** |
| **FST** | 760.241 | 572.521 | 230.230 | 559.959 | 575.533 | 892.903 | 508.860 | 403.103 | 1.31 | 0.71 |  |
| **TIMP2** | 185.649 | 178.783 | 212.782 | 222.184 | 177.055 | 171.974 | 210.389 | 239.400 | 1.00 | 0.06 |  |
| **IGFBP4** | 175.050 | 171.227 | 140.493 | 87.772 | 141.769 | 112.305 | 88.056 | 61.573 | 0.70 | 0.08 |  |
| **SERPINE1** | 98.172 | 101.532 | 96.698 | 106.195 | 90.303 | 98.619 | 93.011 | 114.226 | 0.98 | 0.07 |  |
| **IGFBP6** | 86.903 | 80.689 | 88.036 | 82.835 | 80.494 | 80.176 | 91.585 | 87.589 | 1.00 | 0.06 |  |
| **IL6ST** | 31.798 | 67.477 | 37.234 | 48.169 | 29.031 | 48.658 | 27.620 | 26.835 | 0.73 | 0.15 |  |
| **TIMP1** | 31.296 | 26.876 | 32.273 | 34.493 | 31.652 | 29.379 | 37.295 | 40.253 | 1.11 | 0.07 |  |
| **IL6** | 19.024 | 1.664 | 6.338 | 62.637 | 6.292 | 3.294 | 8.886 | 11.183 | 0.97 | 0.86 |  |
| **CTSS** | 20.251 | 13.506 | 10.799 | 30.214 | 62.102 | 56.805 | 60.541 | 59.348 | **3.71** | 1.56 | 0.0402 |
| **PLG** | 14.679 | 19.831 | 17.747 | 17.649 | 11.041 | 8.138 | 7.950 | 10.726 | 0.55 | 0.16 |  |
| **TNFRSF1a** | 11.455 | 21.271 | 18.277 | 8.871 | 6.137 | 6.366 | 15.704 | 7.202 | 0.63 | 0.26 |  |
| **CCL2** | 14.239 | 9.365 | 11.416 | 16.643 | 14.482 | 14.080 | 17.131 | 18.579 | 1.28 | 0.25 |  |
| **DKK1** | 11.377 | 12.392 | 8.294 | 8.952 | 11.634 | 10.526 | 12.451 | 10.617 | 1.14 | 0.28 |  |
| **IGFBP3** | 6.092 | 11.821 | 7.913 | 14.534 | ND | ND | ND | ND | **L** |  |  |
| **ICAM1** | 9.081 | 4.379 | 5.826 | 12.834 | 22.491 | 20.969 | 42.615 | 29.668 | **4.22** | 2.35 | 0.0713 |
| **IL2RB** | 8.040 | 6.976 | 2.878 | 10.939 | 27.863 | 31.277 | 23.650 | 25.991 | **4.64** | 2.54 | 0.0643 |
| **FLT1** | 3.102 | 5.051 | 10.143 | 4.635 | 3.655 | 8.843 | 21.880 | 6.003 | 1.60 | 0.45 |  |
| **ANG** | 3.812 | 7.500 | 6.337 | 2.369 | 2.009 | 2.790 | 1.563 | 1.104 | **0.40** | 0.12 | 0.0021 |
| **GDF15** | 4.021 | 4.772 | 4.511 | 4.391 | 3.720 | 4.652 | 3.279 | 2.242 | 0.78 | 0.21 |  |
| **OPG** | 3.530 | 2.576 | 8.227 | 2.489 | 1.994 | 1.395 | 8.487 | 1.836 | 0.72 | 0.23 |  |
| **IL1RL1** | 3.185 | 4.314 | 3.408 | 4.585 | 7.592 | 8.125 | 8.031 | 5.229 | 1.94 | 0.58 |  |
| **CXCL5** | 1.633 | 0.173 | 0.296 | 12.906 | 0.082 | 0.042 | 0.017 | 0.022 | **0.09** | 0.11 | 0.0005 |
| **CSF1** | 3.369 | 3.256 | 2.936 | 2.364 | 4.771 | 5.556 | 7.318 | 4.105 | 1.84 | 0.46 |  |
| **SHH** | 2.454 | 3.591 | 1.793 | 2.660 | 3.340 | 3.361 | 2.497 | 1.558 | 1.07 | 0.38 |  |
| **CCL5** | 2.379 | 0.531 | 0.523 | 5.911 | 0.940 | 1.534 | 0.584 | 0.603 | 1.12 | 1.25 |  |
| **PLAUR** | 1.145 | 2.479 | 1.759 | 1.384 | 0.120 | 0.592 | 0.443 | 0.150 | **0.18** | 0.08 | 0.0003 |
| **VEGFA** | 1.561 | 1.017 | 1.376 | 2.512 | 1.486 | 1.817 | 2.282 | 1.618 | 1.26 | 0.55 |  |
| **CXCL8** | 1.948 | 0.367 | 0.602 | 2.961 | 0.444 | 0.198 | 0.149 | 0.231 | **0.27** | 0.19 | 0.0046 |
| **CXCL1** | 2.085 | 0.271 | 0.296 | 2.834 | 0.298 | 0.116 | 0.001 | 0.042 | **0.15** | 0.20 | 0.0034 |
| **TNFRSF1b** | 0.950 | 1.235 | 0.879 | 1.828 | 0.849 | 0.993 | 1.830 | 1.469 | 1.15 | 0.63 |  |
| **AXL** | 1.125 | 1.597 | 1.358 | 0.430 | 1.346 | 1.718 | 1.792 | 0.649 | 1.28 | 0.18 |  |
| **CCL4** | 1.236 | 0.179 | 0.521 | 2.277 | 0.405 | 0.321 | 0.539 | 0.842 | 0.88 | 0.69 |  |
| **CD14** | 0.899 | 1.590 | 0.887 | 0.446 | 1.172 | 1.399 | 0.831 | 0.553 | 1.09 | 0.21 |  |
| **EGFR** | 0.634 | 1.030 | 1.324 | 0.751 | 0.882 | 0.985 | 2.671 | 0.965 | 1.41 | 0.44 |  |
| **TYRO3** | 1.673 | 0.310 | 0.563 | 1.235 | 0.632 | 0.088 | 2.182 | 1.654 | 1.47 | 1.67 |  |
| **HGF** | 0.573 | 0.781 | 1.036 | 1.402 | 0.223 | 0.316 | 0.766 | 0.283 | **0.43** | 0.22 | 0.0140 |
| **CD40** | 0.854 | 0.769 | 0.679 | 0.723 | 0.913 | 0.971 | 0.685 | 0.366 | 0.96 | 0.32 |  |
| **SPP1** | 0.635 | 0.720 | 0.871 | 0.688 | 0.065 | 0.133 | 0.570 | 0.532 | **0.43** | 0.33 | 0.0408 |
| **TREM1** | 0.359 | 0.968 | 0.705 | 0.601 | 0.941 | 1.268 | 0.367 | 0.772 | 1.43 | 0.87 |  |
| **LIF** | 0.650 | 0.234 | 0.912 | 0.477 | 0.139 | 0.501 | 0.508 | 0.744 | 1.12 | 0.89 |  |
| **TGFB1** | 0.465 | 0.922 | 0.432 | 0.384 | 0.332 | 0.458 | 0.179 | 0.331 | 0.62 | 0.20 |  |
| **MIF** | 0.524 | 0.379 | 0.604 | 0.352 | 0.371 | 0.167 | 0.939 | 0.459 | 1.00 | 0.52 |  |
| **CXCL16** | 0.229 | 0.440 | 0.503 | 0.467 | 1.871 | 2.100 | 1.689 | 2.251 | **5.28** | 2.05 | 0.025 |
| **SIGLEC5** | 0.380 | 0.509 | 0.463 | 0.257 | 0.461 | 0.472 | 0.403 | 0.312 | 1.06 | 0.18 |  |
| **FGF7** | 0.372 | 0.549 | 0.360 | 0.318 | 0.012 | 0.247 | 0.154 | 0.067 | **0.28** | 0.20 | 0.0055 |
| **FAS** | 0.270 | 0.261 | 0.309 | 0.286 | 0.446 | 0.450 | 0.573 | 0.337 | 1.60 | 0.30 |  |
| **IL23A** | 0.291 | 0.198 | 0.282 | 0.177 | 0.140 | 0.082 | 0.225 | 0.231 | 0.75 | 0.41 |  |
| **ENG** | 0.216 | 0.072 | 0.343 | 0.081 | 0.060 | 0.035 | 0.924 | 0.152 | 1.33 | 1.15 |  |
| **CCL27** | 0.041 | 0.063 | 0.214 | 0.077 | 0.123 | 0.130 | 0.061 | 0.123 | 1.75 | 1.14 |  |
| **IL2RA** | 0.053 | 0.150 | 0.075 | 0.097 | 0.020 | 0.102 | 0.022 | 0.047 | **0.46** | 0.17 | 0.0079 |
| **IL1B** | 0.118 | 0.018 | 0.019 | 0.212 | ND | ND | ND | ND | **L** |  |  |
| **KITLG** | 0.072 | 0.047 | 0.105 | 0.090 | ND | ND | ND | ND | **L** |  |  |
| **TNFSF14** | 0.134 | 0.021 | 0.096 | 0.044 | ND | ND | ND | ND | **L** |  |  |
| **CCL3** | 0.016 | 0.009 | 0.024 | 0.058 | 0.004 | 0.013 | 0.014 | 0.010 | 0.63 | 0.61 |  |
| **CCL13** | 0.002 | 0.001 | 0.006 | 0.097 | 0.888 | 0.274 | 0.867 | 2.242 | **183.81** | 140.94 | 0.0808 |
| **CXCL12** | 0.022 | 0.041 | 0.017 | 0.013 | ND | ND | ND | ND | **L** |  |  |
| **IL15** | 0.015 | 0.017 | 0.011 | 0.014 | 0.006 | 0.009 | 0.017 | 0.005 | 0.70 | 0.56 |  |
| **CXCL9** | ND | ND | ND | ND | 25.013 | 11.272 | 11.228 | 32.158 | **G** |  |  |
| **CXCL10** | ND | ND | ND | ND | 19.227 | 9.074 | 9.572 | 21.838 | **G** |  |  |
| **CCL8** | ND | ND | ND | ND | 2.913 | 2.257 | 3.178 | 6.478 | **G** |  |  |
| **VCAM1** | ND | ND | ND | ND | 2.234 | 1.774 | 0.683 | 1.001 | **G** |  |  |
| **IFNG** | ND | ND | ND | ND | 0.116 | 0.026 | 0.053 | 0.123 | **G** |  |  |
| **CCL7** | ND | ND | ND | ND | 0.017 | 0.002 | 0.035 | 0.015 | **G** |  |  |

**L stands for Lost and G for Gained with inflammatory priming. ND stands for not detected or below lower limit of ELISA detection.**
