# Supplementary material for: Inflammatory priming enhances mesenchymal stromal cell secretome potential as a clinical product for regenerative medicine approaches through secreted factors and EV-miRNAs: the example of joint disease
Source: Stem Cell Res Ther. 2020 Apr 28;11:165. doi: 10.1186/s13287-020-01677-9 (PMC7189600; doi:10.1186/s13287-020-01677-9)
Supplement: Supplementary file 3 — Additional file 3: Table 3. Expression values and ratios for miRNAs in ASC/iASC EVs. [file 13287_2020_1677_MOESM3_ESM.docx]

|  | **C_RT_ for EVs-embedded miRNAs** | | | | | | | |  |  |  |
| --- | --- | --- | --- | --- | --- | --- | --- | --- | --- | --- | --- |
| **miR** | **EV1** | **EV2** | **EV3** | **EV4** | **iEV1** | **iEV2** | **iEV3** | **iEV4** | **iEV/EV** | **SD** | **p-value** |
| **hsa-miR-24-3p** | 9.44 | 9.43 | 9.26 | 8.95 | 9.38 | 9.70 | 9.73 | 9.24 | 0.85 | 0.13 |  |
| **hsa-miR-125b-5p** | 9.59 | 9.72 | 9.87 | 9.84 | 9.62 | 9.70 | 9.78 | 10.05 | 0.98 | 0.08 |  |
| **hsa-miR-221-3p** | 11.48 | 10.19 | 11.01 | 11.40 | 13.32 | 13.51 | 13.78 | 13.98 | **0.17** | 0.08 | 0.0002 |
| **hsa-miR-222-3p** | 11.19 | 11.21 | 10.82 | 10.95 | 10.65 | 11.11 | 10.70 | 10.71 | 1.20 | 0.18 |  |
| **hsa-miR-21-5p** | 10.98 | 11.09 | 11.32 | 11.43 | 10.99 | 10.68 | 10.72 | 11.49 | 1.20 | 0.27 |  |
| **hsa-miR-193b-3p** | 11.56 | 11.20 | 11.28 | 10.93 | 11.18 | 11.26 | 11.62 | 10.92 | 1.01 | 0.21 |  |
| **hsa-miR-100-5p** | 11.72 | 11.98 | 11.99 | 11.27 | 12.29 | 12.54 | 12.16 | 12.04 | 0.71 | 0.13 |  |
| **hsa-miR-99a-5p** | 11.60 | 12.02 | 11.85 | 11.50 | 12.37 | 12.70 | 12.09 | 12.09 | 0.68 | 0.12 |  |
| **hsa-miR-30c-5p** | 11.96 | 11.92 | 12.22 | 11.77 | 12.08 | 12.42 | 12.20 | 12.68 | 0.79 | 0.22 |  |
| **hsa-miR-30b-5p** | 12.37 | 12.27 | 12.50 | 12.33 | 12.40 | 12.51 | 12.60 | 13.00 | 0.85 | 0.15 |  |
| **hsa-miR-92a-3p** | 13.00 | 12.32 | 12.45 | 12.93 | 12.78 | 12.44 | 13.14 | 12.93 | 0.93 | 0.23 |  |
| **hsa-miR-145-5p** | 12.58 | 13.18 | 12.00 | 12.98 | 13.74 | 14.46 | 13.58 | 14.45 | **0.39** | 0.05 | 0.0002 |
| **hsa-miR-191-5p** | 12.98 | 12.67 | 12.50 | 12.68 | 13.12 | 13.69 | 13.32 | 12.98 | 0.69 | 0.20 |  |
| **hsa-miR-99b-5p** | 12.65 | 13.19 | 12.97 | 12.86 | 12.72 | 12.87 | 12.79 | 12.97 | 1.07 | 0.16 |  |
| **hsa-miR-20a-5p** | 13.42 | 12.53 | 13.26 | 13.46 | 13.49 | 13.17 | 13.22 | 13.63 | 0.88 | 0.17 |  |
| **hsa-miR-31-5p** | 13.46 | 13.84 | 12.75 | 12.69 | 13.24 | 12.75 | 12.06 | 12.63 | 1.49 | 0.50 |  |
| **hsa-miR-19b-3p** | 13.51 | 12.78 | 13.21 | 13.65 | 13.42 | 13.14 | 13.35 | 13.61 | 0.95 | 0.13 |  |
| **hsa-miR-214-3p** | 13.54 | 13.62 | 13.15 | 12.97 | 13.63 | 13.80 | 13.73 | 13.42 | 0.81 | 0.13 |  |
| **hsa-miR-574-3p** | 13.67 | 13.59 | 13.49 | 13.37 | 14.08 | 14.26 | 14.26 | 13.70 | 0.69 | 0.10 |  |
| **hsa-miR-199a-3p** | 13.44 | 13.80 | 13.98 | 13.50 | 13.70 | 13.86 | 13.67 | 13.82 | 0.96 | 0.20 |  |
| **hsa-miR-29a-3p** | 13.93 | 13.48 | 13.55 | 13.92 | 13.12 | 13.06 | 12.90 | 13.31 | 1.55 | 0.17 |  |
| **hsa-miR-27a-3p** | 13.43 | 13.57 | 13.96 | 13.93 | 14.72 | 15.43 | 15.49 | 15.72 | **0.33** | 0.06 | 0.0002 |
| **hsa-miR-218-5p** | 14.12 | 13.22 | 13.74 | 14.54 | 13.81 | 13.54 | 13.65 | 14.43 | 1.04 | 0.18 |  |
| **hsa-miR-127-3p** | 13.81 | 14.17 | 13.81 | 13.88 | 13.62 | 13.93 | 13.84 | 13.81 | 1.09 | 0.09 |  |
| **hsa-miR-34a-5p** | 13.87 | 14.20 | 14.54 | 13.71 | 13.62 | 13.78 | 14.24 | 13.65 | 1.20 | 0.13 |  |
| **hsa-miR-328-3p** | 14.25 | 14.23 | 14.37 | 13.72 | 14.57 | 14.60 | 15.76 | 14.24 | 0.66 | 0.19 |  |
| **hsa-miR-26a-5p** | 14.07 | 14.90 | 13.92 | 13.91 | 13.73 | 13.70 | 13.34 | 13.79 | 1.54 | 0.53 |  |
| **hsa-miR-152-3p** | 14.43 | 14.47 | 14.45 | 14.10 | 14.49 | 14.70 | 14.76 | 14.43 | 0.85 | 0.07 |  |
| **hsa-miR-224-5p** | 14.22 | 14.16 | 13.94 | 15.15 | 14.69 | 14.64 | 14.48 | 15.70 | 0.70 | 0.02 |  |
| **hsa-miR-130a-3p** | 14.40 | 14.02 | 14.57 | 14.52 | 13.95 | 14.26 | 14.23 | 13.90 | 1.26 | 0.30 |  |
| **hsa-miR-320a-3p** | 14.76 | 14.69 | 14.48 | 13.97 | ND | ND | ND | ND | **L** |  |  |
| **hsa-miR-132-3p** | 14.69 | 14.86 | 14.52 | 13.87 | ND | ND | ND | ND | **L** |  |  |
| **hsa-miR-197-3p** | 14.56 | 14.66 | 14.72 | 14.29 | 14.63 | 14.78 | 15.79 | 14.40 | 0.82 | 0.23 |  |
| **hsa-miR-17-5p** | 15.25 | 14.17 | 14.52 | 14.96 | 15.28 | 14.76 | 14.80 | 14.96 | 0.87 | 0.16 |  |
| **hsa-miR-484** | 14.93 | 14.67 | 15.07 | 14.62 | 14.68 | 14.93 | 15.36 | 14.68 | 0.95 | 0.17 |  |
| **hsa-miR-106a-5p** | 15.58 | 14.16 | 14.84 | 14.94 | 15.32 | 14.79 | 14.83 | 15.04 | 0.94 | 0.23 |  |
| **hsa-miR-16-5p** | 14.79 | 14.69 | 15.26 | 14.85 | 14.72 | 14.83 | 14.77 | 15.35 | 1.02 | 0.29 |  |
| **hsa-miR-29c-3p** | 15.08 | 14.84 | 15.05 | 15.16 | 14.01 | 14.31 | 13.92 | 14.06 | 1.96 | 0.35 |  |
| **hsa-miR-27b-3p** | 14.72 | 14.99 | 15.52 | 15.52 | 15.67 | 16.23 | 16.48 | 16.49 | **0.49** | 0.04 | 0.0001 |
| **hsa-miR-106b-5p** | 15.40 | 14.76 | 15.25 | 15.71 | 15.29 | 15.46 | 15.22 | 15.64 | 0.94 | 0.22 |  |
| **hsa-miR-331-3p** | 15.16 | 15.81 | 15.57 | 15.33 | 14.97 | 14.81 | 14.65 | 15.49 | 1.48 | 0.55 |  |
| **hsa-miR-10a-5p** | 15.18 | 16.24 | 15.44 | 15.70 | 15.25 | 15.47 | 14.66 | 15.79 | 1.33 | 0.44 |  |
| **hsa-miR-210-3p** | 16.38 | 15.91 | 15.29 | 15.06 | 14.79 | 14.50 | 14.55 | 14.38 | **2.23** | 0.71 | 0.0405 |
| **hsa-miR-30a-5p** | 15.29 | 15.84 | 16.04 | 15.52 | 15.28 | 15.23 | 14.87 | 15.87 | 1.39 | 0.65 |  |
| **hsa-miR-376a-3p** | 15.61 | 15.20 | 15.62 | 16.31 | 15.72 | 16.18 | 16.06 | 16.86 | 0.71 | 0.17 |  |
| **hsa-let-7a-5p** | 15.42 | 17.59 | 15.52 | 14.66 | 14.85 | 14.84 | 13.83 | 14.24 | 3.19 | 2.50 | 0.1781 |
| **hsa-miR-138-5p** | 16.31 | 16.18 | 16.48 | 14.74 | 15.69 | 16.88 | 16.34 | 14.85 | 1.04 | 0.38 |  |
| **hsa-miR-193a-5p** | 16.36 | 15.93 | 15.60 | 15.92 | 16.32 | 15.98 | 16.35 | 15.97 | 0.89 | 0.20 |  |
| **hsa-miR-296-5p** | 15.93 | 15.88 | 16.25 | 15.90 | 16.27 | 16.29 | 16.84 | 16.50 | 0.72 | 0.07 |  |
| **hsa-miR-25-3p** | 16.24 | 15.16 | 16.10 | 16.71 | 16.32 | 15.88 | 16.03 | 16.61 | 0.92 | 0.21 |  |
| **hsa-let-7c-5p** | 15.50 | 16.18 | 15.83 | 16.95 | 15.23 | 14.94 | 15.14 | 15.47 | 1.99 | 0.71 |  |
| **hsa-miR-22-3p** | 16.32 | 16.09 | 15.77 | 16.40 | 16.32 | 16.43 | 15.76 | 16.34 | 0.96 | 0.11 |  |
| **hsa-miR-409-3p** | 15.90 | 16.85 | 16.19 | 15.73 | 16.07 | 16.53 | 16.43 | 16.67 | 0.88 | 0.30 |  |
| **hsa-miR-365a-3p** | 16.38 | 15.95 | 15.85 | 16.56 | 16.70 | 15.93 | 16.13 | 16.88 | 0.86 | 0.10 |  |
| **hsa-miR-376c-3p** | 16.32 | 15.89 | 16.21 | 16.64 | 16.24 | 16.56 | 16.02 | 17.05 | 0.89 | 0.24 |  |
| **hsa-miR-26b-5p** | 16.17 | 16.69 | 16.48 | 16.03 | 16.19 | 16.04 | 15.83 | 15.97 | 1.29 | 0.32 |  |
| **hsa-miR-149-5p** | 16.50 | 16.55 | 16.28 | 16.72 | 18.86 | 19.15 | 19.79 | 19.79 | **0.14** | 0.05 | 0.0001 |
| **hsa-miR-28-5p** | 16.50 | 16.70 | 16.42 | 16.43 | 15.69 | 15.74 | 15.48 | 16.01 | 1.74 | 0.28 |  |
| **hsa-miR-31-3p** | 16.48 | 16.73 | 16.75 | 16.15 | 17.21 | 17.20 | 16.72 | 16.83 | 0.74 | 0.19 |  |
| **hsa-miR-28-3p** | 16.87 | 16.61 | 16.25 | 16.66 | 16.21 | 16.70 | 15.75 | 16.38 | 1.29 | 0.28 |  |
| **hsa-miR-483-5p** | 16.53 | 16.83 | 17.26 | 15.95 | 15.62 | 17.05 | 18.32 | 16.45 | 0.98 | 0.62 |  |
| **hsa-miR-195-5p** | 16.47 | 16.62 | 17.04 | 16.79 | 16.22 | 16.37 | 16.55 | 16.99 | 1.16 | 0.22 |  |
| **hsa-miR-143-3p** | 16.50 | 17.24 | 16.43 | 16.80 | 16.86 | 17.58 | 16.25 | 17.79 | 0.80 | 0.26 |  |
| **hsa-miR-15b-5p** | 16.65 | 16.83 | 16.55 | 17.08 | 16.54 | 16.70 | 15.98 | 16.91 | 1.20 | 0.19 |  |
| **hsa-miR-181a-5p** | 16.74 | 18.08 | 16.48 | 15.92 | 16.73 | 17.01 | 16.01 | 14.94 | 1.61 | 0.51 |  |
| **hsa-miR-370-3p** | 16.89 | 16.75 | 16.74 | 16.91 | 16.60 | 17.07 | 17.22 | 16.96 | 0.93 | 0.22 |  |
| **hsa-miR-335-5p** | 17.53 | 17.12 | 16.04 | 16.91 | 18.20 | 17.33 | 16.82 | 17.56 | 0.68 | 0.13 |  |
| **hsa-miR-1260a** | 16.53 | 17.55 | 17.03 | 16.76 | 18.13 | 16.42 | 17.40 | 16.97 | 1.04 | 0.80 |  |
| **hsa-miR-361-5p** | 17.08 | 16.72 | 17.03 | 17.04 | 17.71 | 17.97 | 17.71 | 18.31 | 0.53 | 0.13 |  |
| **hsa-miR-23a-3p** | 16.86 | 16.82 | 17.34 | 17.02 | 17.32 | 17.92 | 17.80 | 17.83 | 0.62 | 0.13 |  |
| **hsa-miR-532-5p** | 17.47 | 17.03 | 17.12 | 16.94 | 17.60 | 17.72 | 17.64 | 17.21 | 0.76 | 0.13 |  |
| **hsa-miR-148a-3p** | 17.29 | 16.71 | 17.03 | 17.77 | 17.21 | 17.08 | 17.32 | 17.92 | 0.89 | 0.13 |  |
| **hsa-miR-886-3p** | 17.49 | 17.22 | 16.43 | 17.68 | 16.24 | 15.65 | 15.66 | 16.08 | **2.52** | 0.62 | 0.0162 |
| **hsa-miR-29b-3p** | 17.44 | 17.30 | 17.51 | 17.11 | 15.70 | 15.77 | 15.10 | 15.87 | **3.47** | 1.30 | 0.032 |
| **hsa-miR-342-3p** | 17.78 | 17.81 | 17.15 | 17.00 | 17.65 | 18.27 | 17.73 | 17.53 | 0.80 | 0.20 |  |
| **hsa-miR-130b-3p** | 17.40 | 16.82 | 17.36 | 18.18 | 17.27 | 17.42 | 17.81 | 17.92 | 0.92 | 0.27 |  |
| **hsa-miR-660-5p** | 17.72 | 17.16 | 17.49 | 17.44 | 17.98 | 17.96 | 17.74 | 17.41 | 0.82 | 0.18 |  |
| **hsa-miR-663b** | 17.63 | 16.76 | 17.45 | 18.09 | 18.69 | 18.20 | 19.57 | 17.65 | 0.61 | 0.51 |  |
| **hsa-miR-324-5p** | 17.49 | 17.55 | 17.45 | 17.58 | 17.67 | 17.70 | 17.74 | 18.00 | 0.84 | 0.07 |  |
| **hsa-miR-93-5p** | 17.67 | 17.11 | 17.48 | 17.86 | 18.45 | 18.13 | 18.08 | 18.24 | 0.63 | 0.12 |  |
| **hsa-miR-654-5p** | 18.20 | 17.54 | 17.29 | 17.28 | ND | ND | ND | ND |  |  |  |
| **hsa-miR-374a-5p** | 17.73 | 18.37 | 18.39 | 17.50 | 17.82 | 17.74 | 16.79 | 18.07 | 1.55 | 1.05 |  |
| **hsa-miR-30a-3p** | 17.87 | 18.57 | 17.09 | 18.57 | 18.95 | 19.42 | 18.53 | 18.89 | 0.55 | 0.18 |  |
| **hsa-miR-886-5p** | 18.55 | 18.49 | 17.16 | 17.91 | 18.00 | 17.76 | 16.98 | 17.63 | 1.37 | 0.24 |  |
| **hsa-miR-30e-3p** | 17.55 | 18.41 | 18.25 | 18.08 | 19.75 | 18.96 | 18.74 | 18.71 | 0.57 | 0.23 |  |
| **hsa-miR-10b-5p** | 17.81 | 19.12 | 18.21 | 17.55 | 17.65 | 18.33 | 16.76 | 17.76 | 1.61 | 0.83 |  |
| **hsa-miR-140-5p** | 18.28 | 18.28 | 18.27 | 17.99 | 18.52 | 18.51 | 17.14 | 18.10 | 1.20 | 0.65 |  |
| **hsa-miR-30d-5p** | 17.62 | 18.32 | 18.76 | 18.18 | 17.66 | 18.24 | 17.68 | 18.61 | 1.22 | 0.61 |  |
| **hsa-miR-495-3p** | 18.28 | 17.59 | 18.02 | 19.02 | 18.41 | 18.58 | 18.50 | 19.09 | 0.77 | 0.21 |  |
| **hsa-miR-146b-5p** | 18.65 | 18.76 | 17.61 | 17.98 | 14.90 | 14.75 | 14.86 | 14.28 | **12.33** | 4.00 | 0.0109 |
| **hsa-miR-411-5p** | 18.01 | 18.75 | 18.35 | 18.04 | 17.31 | 17.57 | 16.78 | 17.58 | **2.06** | 0.71 | 0.0583 |
| **hsa-let-7g-5p** | 18.17 | 18.86 | 18.15 | 17.98 | 18.14 | 18.05 | 17.76 | 18.27 | 1.23 | 0.41 |  |
| **hsa-miR-34c-5p** | 17.88 | 17.93 | 19.40 | 18.03 | 18.04 | 17.99 | 19.53 | 18.81 | 0.84 | 0.17 |  |
| **hsa-miR-199b-5p** | 18.17 | 18.20 | 18.47 | 18.67 | ND | ND | ND | ND |  |  |  |
| **hsa-miR-186-5p** | 18.76 | 18.26 | 18.39 | 18.56 | 19.14 | 19.15 | 18.70 | 18.79 | 0.74 | 0.14 |  |
| **hsa-miR-423-5p** | 18.66 | 18.23 | 18.52 | 18.63 | 18.62 | 18.45 | 19.11 | 18.72 | 0.87 | 0.16 |  |
| **hsa-miR-34a-3p** | 18.10 | 18.62 | 19.11 | 18.23 | 18.98 | 19.39 | 18.83 | 18.50 | 0.80 | 0.31 |  |
| **hsa-miR-744-5p** | 18.59 | 19.09 | 18.24 | 18.18 | 18.98 | 19.63 | 19.15 | 18.40 | 0.71 | 0.14 |  |
| **hsa-miR-204-5p** | 17.49 | 19.61 | 19.30 | 17.97 | 17.65 | 19.33 | 19.80 | 17.94 | 0.96 | 0.22 |  |
| **hsa-miR-19a-3p** | 18.87 | 18.08 | 18.45 | 19.00 | 18.61 | 18.36 | 18.65 | 18.90 | 0.99 | 0.17 |  |
| **hsa-miR-212-3p** | 19.00 | 18.83 | 18.69 | 18.05 | 18.47 | 18.64 | 18.83 | 18.15 | 1.11 | 0.25 |  |
| **hsa-miR-125a-5p** | 18.27 | 18.36 | 19.14 | 18.95 | 19.04 | 18.90 | 19.33 | 19.82 | 0.68 | 0.15 |  |
| **hsa-miR-374b-5p** | 18.53 | 18.73 | 18.91 | 18.68 | 18.57 | 18.44 | 17.01 | 18.98 | 1.69 | 1.38 |  |
| **hsa-miR-424-5p** | 18.03 | 19.11 | 19.23 | 18.66 | 18.25 | 17.75 | 17.50 | 18.59 | 1.95 | 1.19 |  |
| **hsa-miR-199a-5p** | 18.61 | 18.89 | 18.89 | 18.79 | 20.00 | 19.80 | 20.05 | 20.41 | **0.42** | 0.09 | 0.001 |
| **hsa-miR-10b-3p** | 18.84 | 19.11 | 18.75 | 18.53 | 19.23 | 19.40 | 18.81 | 18.70 | 0.86 | 0.08 |  |
| **hsa-miR-1291** | 18.51 | 19.11 | 18.95 | 18.89 | 18.72 | 19.21 | 18.62 | 19.15 | 0.97 | 0.19 |  |
| **hsa-miR-103a-3p** | 18.73 | 19.48 | 18.58 | 18.69 | 18.54 | 18.51 | 16.80 | 18.23 | 1.98 | 1.03 |  |
| **hsa-miR-29a-5p** | 18.67 | 18.98 | 19.27 | 18.81 | 18.74 | 18.56 | 18.60 | 18.25 | 1.34 | 0.28 |  |
| **hsa-miR-301a-3p** | 19.09 | 18.70 | 18.75 | 19.44 | 18.86 | 19.55 | 18.92 | 18.91 | 1.02 | 0.38 |  |
| **hsa-miR-532-3p** | 19.30 | 19.00 | 19.02 | 18.67 | 19.43 | 19.61 | 19.42 | 19.00 | 0.78 | 0.11 |  |
| **hsa-miR-410-3p** | 18.61 | 18.76 | 19.22 | 19.48 | 18.81 | 19.30 | 18.79 | 19.94 | 0.91 | 0.30 |  |
| **hsa-miR-7-1-3p** | 19.13 | 19.05 | 19.04 | 19.05 | 19.34 | 19.23 | 18.73 | 19.24 | 0.97 | 0.18 |  |
| **hsa-miR-134-5p** | 19.18 | 18.70 | 19.16 | 19.35 | 18.70 | 19.45 | 19.71 | 19.07 | 0.97 | 0.39 |  |
| **hsa-miR-494-3p** | 19.06 | 18.86 | 18.97 | 19.53 | ND | ND | ND | ND | **L** |  |  |
| **hsa-miR-137-3p** | 19.59 | 19.15 | 19.18 | 19.01 | 19.68 | 19.40 | 18.52 | 18.76 | 1.14 | 0.33 |  |
| **hsa-miR-590-5p** | 19.48 | 19.08 | 19.46 | 19.76 | 19.19 | 19.45 | 19.21 | 19.07 | 1.20 | 0.34 |  |
| **hsa-miR-503-5p** | 19.48 | 19.38 | 19.40 | 19.60 | 20.60 | 20.09 | 20.84 | 20.53 | **0.49** | 0.10 | 0.0020 |
| **hsa-miR-379-5p** | 19.48 | 20.45 | 19.79 | 19.99 | 19.03 | 19.13 | 18.51 | 19.89 | 1.84 | 0.73 |  |
| **hsa-miR-22-5p** | 19.58 | 20.21 | 20.10 | 19.88 | 19.66 | 20.14 | 19.64 | 19.67 | 1.13 | 0.19 |  |
| **hsa-miR-34b-3p** | 19.49 | 19.46 | 21.08 | 19.91 | 19.76 | 19.83 | 19.92 | 19.58 | 1.27 | 0.68 |  |
| **hsa-let-7d-5p** | 19.57 | 21.11 | 19.79 | 19.69 | 19.20 | 18.99 | 17.68 | 18.87 | **2.93** | 1.62 | 0.0974 |
| **hsa-miR-452-5p** | 19.95 | 20.16 | 19.66 | 20.40 | 20.15 | 20.02 | 19.78 | 20.65 | 0.93 | 0.12 |  |
| **hsa-miR-324-3p** | 20.29 | 19.95 | 20.14 | 20.36 | 20.90 | 20.70 | 20.31 | 20.65 | 0.74 | 0.14 |  |
| **hsa-miR-424-3p** | 20.25 | 20.56 | 19.86 | 20.22 | 20.52 | 19.88 | 19.49 | 20.77 | 1.10 | 0.42 |  |
| **hsa-miR-95-3p** | 18.95 | 18.98 | 20.79 | 22.19 | 18.71 | 18.79 | 20.83 | 21.06 | 1.37 | 0.55 |  |
| **hsa-miR-455-3p** | 20.72 | 21.07 | 19.67 | 20.23 | 19.49 | 19.74 | 19.01 | 19.67 | 1.98 | 0.53 |  |
| **hsa-miR-708-5p** | 20.52 | 20.24 | 21.96 | 19.03 | 20.57 | 19.82 | 21.41 | 18.78 | 1.24 | 0.21 |  |
| **hsa-miR-330-3p** | 21.17 | 20.47 | 19.61 | 20.57 | ND | ND | ND | ND | **L** |  |  |
| **hsa-miR-345-5p** | 20.43 | 20.44 | 19.95 | 21.09 | 21.41 | 21.56 | 21.47 | 21.82 | **0.48** | 0.11 | 0.0025 |
| **hsa-miR-151a-3p** | 20.57 | 20.02 | 20.95 | 20.59 | ND | ND | ND | ND | **L** |  |  |
| **hsa-miR-128-3p** | 20.55 | 19.98 | 20.76 | 21.24 | 21.45 | 21.39 | 21.22 | 21.72 | 0.59 | 0.17 |  |
| **hsa-miR-491-5p** | 20.85 | 21.01 | 21.05 | 19.91 | 20.59 | 20.68 | 20.76 | 19.84 | 1.18 | 0.09 |  |
| **hsa-miR-185-5p** | 20.95 | 20.26 | 20.53 | 21.08 | 21.59 | 21.69 | 21.58 | 21.04 | 0.63 | 0.29 |  |
| **hsa-miR-222-5p** | 20.82 | 20.42 | 21.09 | 20.80 | 19.52 | 19.21 | 20.97 | 20.26 | 1.83 | 0.66 |  |
| **hsa-miR-323-3p** | 21.00 | 20.22 | 20.75 | 21.30 | 20.52 | 20.99 | 21.49 | 21.40 | 0.88 | 0.38 |  |
| **hsa-miR-664-3p** | 20.98 | 21.84 | 20.67 | 20.41 | ND | ND | ND | ND | **L** |  |  |
| **hsa-miR-485-3p** | 20.70 | 21.09 | 21.14 | 20.98 | 20.32 | 21.11 | 21.54 | 21.03 | 1.00 | 0.23 |  |
| **hsa-miR-18a-5p** | 21.53 | 20.24 | 21.17 | 21.41 | 21.56 | 20.98 | 21.61 | 21.81 | 0.77 | 0.16 |  |
| **hsa-miR-15a-5p** | 21.19 | 21.54 | 21.57 | 20.20 | 20.68 | 20.60 | 20.58 | 20.12 | 1.60 | 0.44 |  |
| **hsa-miR-193b-5p** | 21.31 | 21.54 | 21.46 | 20.52 | 21.46 | 21.02 | 21.28 | 20.95 | 1.05 | 0.30 |  |
| **hsa-miR-155-5p** | 21.95 | 21.60 | 20.09 | 21.18 | 20.50 | 19.68 | 18.72 | 20.26 | **2.75** | 0.78 | 0.0206 |
| **hsa-miR-487b-3p** | 21.21 | 21.08 | 20.94 | 21.87 | 21.72 | 21.87 | 21.40 | 23.07 | 0.61 | 0.13 |  |
| **hsa-miR-184** | 21.08 | 22.17 | 21.64 | 20.38 | 21.42 | 21.97 | 22.22 | 21.17 | 0.79 | 0.25 |  |
| **hsa-miR-433-3p** | 21.35 | 21.63 | 21.36 | 21.02 | 21.13 | 20.73 | 21.70 | 21.45 | 1.14 | 0.52 |  |
| **hsa-miR-455-5p** | 21.97 | 21.13 | 20.66 | 21.61 | 19.85 | 19.60 | 19.56 | 19.59 | **3.35** | 1.02 | 0.0192 |
| **hsa-miR-148b-3p** | 21.36 | 21.18 | 21.58 | 21.63 | 21.19 | 20.77 | 21.48 | 21.46 | 1.16 | 0.11 |  |
| **hsa-miR-339-3p** | 21.51 | 21.23 | 21.15 | 21.93 | 22.02 | 23.11 | 22.66 | 21.82 | 0.60 | 0.37 |  |
| **hsa-miR-337-5p** | 21.41 | 20.96 | 21.50 | 21.99 | 22.10 | 21.76 | 21.81 | 22.44 | 0.68 | 0.11 |  |
| **hsa-miR-539-5p** | 21.34 | 21.45 | 21.37 | 21.72 | 21.42 | 21.62 | 20.31 | 22.17 | 1.16 | 0.62 |  |
| **hsa-miR-154-5p** | 21.47 | 21.61 | 22.11 | 21.04 | ND | ND | ND | ND | **L** |  |  |
| **hsa-miR-543** | 21.40 | 21.66 | 21.65 | 21.82 | 21.75 | 21.39 | 21.96 | 21.66 | 0.98 | 0.21 |  |
| **hsa-miR-146a-5p** | 23.80 | 21.32 | 20.24 | 21.44 | 23.48 | 22.22 | 20.83 | 21.31 | 0.88 | 0.34 |  |
| **hsa-miR-766-3p** | 22.58 | 20.95 | 21.52 | 21.88 | 21.60 | 20.95 | 21.85 | 21.84 | 1.20 | 0.52 |  |
| **hsa-miR-24-2-5p** | 21.76 | 21.93 | 21.90 | 21.45 | 22.42 | 23.19 | 21.94 | 21.62 | 0.73 | 0.25 |  |
| **hsa-miR-638** | 21.58 | 21.98 | 22.09 | 21.47 | 21.31 | 20.71 | 21.98 | 21.03 | 1.51 | 0.61 |  |
| **hsa-miR-140-3p** | 22.01 | 22.01 | 21.25 | 21.98 | 21.74 | 23.16 | 22.19 | 21.01 | 1.03 | 0.70 |  |
| **hsa-miR-203a-3p** | 23.17 | 22.28 | 20.95 | 21.33 | 20.67 | 21.49 | 20.77 | 21.82 | 2.30 | 2.27 | 0.3351 |
| **hsa-miR-505-3p** | 22.53 | 21.22 | 22.07 | 21.91 | 22.35 | 21.03 | 21.78 | 21.85 | 1.14 | 0.07 |  |
| **hsa-miR-942-5p** | 21.49 | 21.90 | 21.99 | 22.40 | 21.75 | 22.20 | 21.78 | 22.36 | 0.96 | 0.17 |  |
| **hsa-miR-381-3p** | 22.18 | 21.39 | 22.27 | 21.98 | 21.76 | 21.91 | 23.41 | 23.25 | 0.73 | 0.43 |  |
| **hsa-miR-125b-1-3p** | 22.22 | 21.99 | 22.19 | 21.65 | 22.54 | 22.47 | 22.59 | 21.88 | 0.78 | 0.06 |  |
| **hsa-miR-502-3p** | 22.31 | 21.35 | 22.15 | 22.75 | 22.45 | 22.39 | 22.32 | 22.35 | 0.90 | 0.34 |  |
| **hsa-miR-139-5p** | 22.04 | 22.54 | 22.31 | 21.77 | 21.81 | 21.90 | 21.78 | 21.90 | 1.27 | 0.29 |  |
| **hsa-miR-500a-5p** | 22.33 | 22.22 | 22.37 | 21.85 | 22.11 | 22.39 | 21.19 | 20.90 | 1.56 | 0.65 |  |
| **hsa-miR-432-5p** | 21.66 | 22.64 | 22.27 | 22.40 | 22.89 | 23.14 | 22.70 | 22.43 | 0.71 | 0.23 |  |
| **hsa-miR-190a-5p** | 21.60 | 23.09 | 22.31 | 22.01 | 21.58 | 22.40 | 19.72 | 20.95 | 2.69 | 2.27 | 0.2332 |
| **hsa-miR-671-3p** | 22.19 | 21.75 | 22.21 | 22.99 | 23.03 | 23.36 | 23.77 | 24.73 | **0.38** | 0.12 | 0.0019 |
| **hsa-miR-20b-5p** | 23.34 | 20.67 | 22.68 | 22.71 | 22.16 | 21.45 | 21.46 | 21.87 | 1.74 | 0.81 |  |
| **hsa-miR-542-3p** | 22.18 | 22.52 | 22.32 | 22.68 | 21.90 | 22.05 | 21.65 | 22.75 | 1.29 | 0.27 |  |
| **hsa-miR-889-3p** | 22.61 | 21.71 | 22.36 | 23.77 | 22.53 | 22.55 | 22.83 | 23.55 | 0.87 | 0.28 |  |
| **hsa-miR-652-3p** | 22.42 | 22.64 | 22.43 | 22.99 | 22.98 | 23.19 | 22.04 | 22.67 | 0.98 | 0.35 |  |
| **hsa-miR-1290** | 22.25 | 22.64 | 22.75 | 23.00 | 22.79 | 23.58 | 22.51 | 22.53 | 0.94 | 0.41 |  |
| **hsa-miR-101-3p** | 22.54 | 22.72 | 22.99 | 22.41 | 22.44 | 22.36 | 21.92 | 22.73 | 1.31 | 0.56 |  |
| **hsa-miR-214-5p** | 22.33 | 22.36 | 23.25 | 22.84 | ND | ND | ND | ND | **L** |  |  |
| **hsa-miR-597-5p** | 22.85 | 22.06 | 22.77 | 23.11 | 23.05 | 22.49 | 23.09 | 22.77 | 0.92 | 0.23 |  |
| **hsa-miR-181c-5p** | 23.03 | 23.22 | 22.18 | 22.55 | 23.17 | 22.21 | 21.59 | 21.78 | 1.53 | 0.46 |  |
| **hsa-miR-1271-5p** | 22.98 | 21.99 | 22.92 | 23.37 | ND | ND | ND | ND | **L** |  |  |
| **hsa-miR-642a-5p** | 22.62 | 21.95 | 22.42 | 24.29 | 22.04 | 21.84 | 22.36 | 23.63 | 1.30 | 0.28 |  |
| **hsa-miR-502-5p** | 22.99 | 23.19 | 22.85 | 22.46 | 22.25 | 22.66 | 21.49 | 21.63 | 1.86 | 0.49 |  |
| **hsa-miR-493-3p** | 22.59 | 22.81 | 22.66 | 23.48 | 23.59 | 23.50 | 23.49 | 24.01 | 0.59 | 0.08 |  |
| **hsa-miR-625-3p** | 22.58 | 22.87 | 23.09 | 23.24 | 23.39 | 23.33 | 23.49 | 23.43 | 0.73 | 0.12 |  |
| **hsa-miR-645** | 21.77 | 23.26 | 23.86 | 22.97 | 22.85 | 22.90 | 24.17 | 22.84 | 0.91 | 0.35 |  |
| **hsa-miR-576-3p** | 22.84 | 22.21 | 22.98 | 23.84 | 23.12 | 22.77 | 23.21 | 23.84 | 0.84 | 0.13 |  |
| **hsa-miR-150-5p** | 24.61 | 22.68 | 22.81 | 22.00 | 22.50 | 19.38 | 23.51 | 22.03 | 3.93 | 4.26 | 0.2627 |
| **hsa-miR-93-3p** | 23.37 | 23.25 | 22.71 | 22.80 | 23.72 | 24.42 | 23.51 | 24.34 | 0.54 | 0.19 |  |
| **hsa-miR-769-5p** | 22.92 | 23.54 | 23.46 | 22.49 | ND | ND | ND | ND | **L** |  |  |
| **hsa-miR-758-3p** | 22.91 | 22.42 | 23.42 | 23.76 | 23.61 | 22.48 | 24.10 | 24.03 | 0.76 | 0.17 |  |
| **hsa-miR-223-3p** | 24.11 | 23.92 | 23.11 | 21.37 | 22.22 | 22.39 | 23.28 | 21.30 | 2.13 | 1.39 | 0.2024 |
| **hsa-miR-136-3p** | 22.92 | 23.15 | 23.50 | 23.12 | 23.53 | 23.65 | 23.15 | 23.73 | 0.82 | 0.30 |  |
| **hsa-miR-99b-3p** | 23.20 | 23.84 | 23.42 | 22.34 | 23.44 | 24.10 | 24.08 | 22.61 | 0.79 | 0.10 |  |
| **hsa-miR-497-5p** | 22.80 | 22.48 | 24.04 | 23.48 | 23.67 | 22.48 | 23.82 | 24.12 | 0.84 | 0.29 |  |
| **hsa-miR-572** | 23.24 | 23.35 | 23.59 | 22.75 | 23.23 | 22.88 | 24.34 | 22.65 | 1.02 | 0.33 |  |
| **hsa-miR-409-5p** | 23.12 | 23.62 | 23.11 | 23.20 | 23.45 | 22.71 | 23.96 | 23.37 | 1.03 | 0.59 |  |
| **hsa-miR-362-5p** | 23.44 | 23.89 | 23.28 | 22.61 | 23.68 | 23.68 | 21.72 | 22.95 | 1.43 | 1.02 |  |
| **hsa-miR-196b-5p** | 22.70 | 24.13 | 23.12 | 23.34 | 22.63 | 23.79 | 22.60 | 23.11 | 1.23 | 0.16 |  |
| **hsa-miR-301b-3p** | 23.59 | 23.42 | 23.70 | 22.58 | 23.93 | 23.72 | 24.27 | 23.68 | 0.69 | 0.16 |  |
| **hsa-miR-299-5p** | 23.08 | 23.33 | 23.41 | 23.52 | ND | ND | ND | ND | **L** |  |  |
| **hsa-miR-329-3p** | 22.57 | 22.68 | 23.38 | 24.78 | 23.31 | 22.66 | 23.42 | 23.56 | 1.23 | 0.76 |  |
| **hsa-miR-369-3p** | 22.24 | 22.84 | 23.68 | 24.72 | 22.95 | 22.70 | 22.76 | 24.19 | 1.26 | 0.54 |  |
| **hsa-miR-126-3p** | 24.20 | 24.49 | 22.67 | 22.19 | 24.64 | 23.77 | 21.75 | 22.10 | 1.33 | 0.53 |  |
| **hsa-miR-1226-5p** | 23.68 | 23.06 | 23.85 | 23.15 | ND | ND | ND | ND | **L** |  |  |
| **hsa-miR-629-3p** | 23.59 | 23.02 | 23.47 | 23.89 | 23.79 | 24.24 | 23.77 | 24.51 | 0.69 | 0.20 |  |
| **hsa-miR-548b-3p** | 23.97 | 22.38 | 23.96 | 23.76 | 22.07 | 23.28 | 26.18 | 24.23 | 1.30 | 1.63 |  |
| **hsa-miR-376b-3p** | 23.27 | 23.46 | 23.89 | 23.57 | 22.68 | 23.38 | 23.01 | 23.56 | 1.35 | 0.39 |  |
| **hsa-miR-194-5p** | 23.85 | 23.82 | 23.22 | 23.64 | 22.69 | 22.77 | 21.01 | 22.82 | **2.67** | 1.31 | 0.084 |
| **hsa-miR-1180-3p** | 23.26 | 23.48 | 23.79 | 24.08 | ND | ND | ND | ND | **L** |  |  |
| **hsa-miR-198** | 22.95 | 25.12 | 23.16 | 23.50 | 21.60 | 22.79 | 24.17 | 21.87 | 2.79 | 1.86 | 0.1499 |
| **hsa-miR-34b-5p** | 23.21 | 23.54 | 24.84 | 23.19 | 23.56 | 22.93 | 24.14 | 23.04 | 1.26 | 0.39 |  |
| **hsa-miR-362-3p** | 23.56 | 22.96 | 23.45 | 24.95 | 24.56 | 24.57 | 24.33 | 24.12 | 0.79 | 0.67 |  |
| **hsa-miR-579-3p** | 23.57 | 22.81 | 24.14 | 24.62 | ND | ND | ND | ND | **L** |  |  |
| **hsa-miR-151a-5p** | 23.43 | 24.26 | 23.82 | 23.88 | 23.87 | 23.53 | 22.54 | 23.87 | 1.46 | 0.75 |  |
| **hsa-miR-296-3p** | 24.10 | 23.43 | 24.23 | 24.35 | ND | ND | ND | ND | **L** |  |  |
| **hsa-miR-616-3p** | 24.59 | 23.23 | 23.69 | 24.88 | ND | ND | ND | ND | **L** |  |  |
| **hsa-miR-27a-5p** | 23.91 | 24.02 | 24.48 | 23.99 | 24.27 | 23.30 | 24.17 | 23.95 | 1.17 | 0.37 |  |
| **hsa-miR-542-5p** | 24.54 | 24.03 | 24.16 | 23.69 | 24.64 | 23.62 | 24.69 | 23.69 | 0.99 | 0.26 |  |
| **hsa-miR-548d-5p** | 24.73 | 23.30 | 24.47 | 23.99 | 24.48 | 23.45 | 26.08 | 24.26 | 0.81 | 0.36 |  |
| **hsa-miR-605-5p** | 24.51 | 23.69 | 24.22 | 24.71 | 24.45 | 23.25 | 26.60 | 24.87 | 0.87 | 0.49 |  |
| **hsa-miR-99a-3p** | 23.88 | 23.94 | 24.59 | 24.89 | 24.60 | 24.58 | 25.04 | 24.19 | 0.90 | 0.49 |  |
| **hsa-miR-146b-3p** | 25.02 | 24.62 | 23.96 | 24.09 | 22.30 | 21.80 | 22.80 | 21.49 | **5.48** | 2.19 | 0.0264 |
| **hsa-miR-654-3p** | 24.54 | 23.51 | 24.72 | 25.01 | ND | ND | ND | ND | **L** |  |  |
| **hsa-miR-27b-5p** | 23.63 | 25.49 | 25.02 | 23.74 | 24.49 | 25.02 | 24.60 | 23.97 | 1.03 | 0.40 |  |
| **hsa-miR-181a-2-3p** | 24.68 | 25.81 | 24.39 | 23.27 | 25.41 | 25.89 | 23.58 | 24.71 | 0.92 | 0.60 |  |
| **hsa-miR-451a** | 24.61 | 24.07 | 25.57 | 24.50 | 23.75 | 24.67 | 26.03 | 23.13 | 1.45 | 0.92 |  |
| **hsa-miR-628-5p** | 25.17 | 25.14 | 24.51 | 24.02 | 24.79 | 24.19 | 23.73 | 24.40 | 1.43 | 0.52 |  |
| **hsa-miR-1255b-5p** | 25.20 | 23.40 | 24.99 | 25.90 | 25.65 | 23.78 | 27.56 | 25.25 | 0.81 | 0.57 |  |
| **hsa-miR-380-5p** | 24.56 | 24.72 | 24.90 | 25.36 | ND | ND | ND | ND | **L** |  |  |
| **hsa-miR-450a-5p** | 24.64 | 25.01 | 25.39 | 24.85 | 25.12 | 23.87 | 24.44 | 24.55 | 1.52 | 0.68 |  |
| **hsa-miR-145-3p** | 24.27 | 25.52 | 24.83 | 25.37 | ND | ND | ND | ND | **L** |  |  |
| **hsa-miR-425-3p** | 25.51 | 25.61 | 25.19 | 23.87 | ND | ND | ND | ND | **L** |  |  |
| **hsa-miR-598-3p** | 26.29 | 25.14 | 24.48 | 24.53 | 25.31 | 23.98 | 25.87 | 24.25 | 1.45 | 0.83 |  |
| **hsa-miR-885-5p** | 26.14 | 24.85 | 25.39 | 24.35 | 24.56 | 23.77 | 26.67 | 25.02 | 1.54 | 1.23 |  |
| **hsa-miR-337-3p** | 25.03 | 24.88 | 25.60 | 25.34 | ND | ND | ND | ND | **L** |  |  |
| **hsa-miR-505-5p** | 25.33 | 25.91 | 25.14 | 25.92 | 25.65 | 25.05 | 25.16 | 24.81 | 1.44 | 0.66 |  |
| **hsa-miR-744-3p** | 25.98 | 25.47 | 25.05 | 26.07 | ND | ND | ND | ND | **L** |  |  |
| **hsa-miR-7-5p** | 26.03 | 25.07 | 25.57 | 25.94 | 24.83 | 24.90 | 24.93 | 24.39 | 1.98 | 0.81 |  |
| **hsa-miR-10a-3p** | 26.52 | 25.89 | 26.06 | 25.88 | ND | ND | ND | ND | **L** |  |  |
| **hsa-miR-30d-3p** | 26.75 | 26.43 | 27.03 | 25.78 | ND | ND | ND | ND | **L** |  |  |
| **hsa-miR-148b-5p** | 26.52 | 26.02 | 27.73 | 25.87 | ND | ND | ND | ND | **L** |  |  |
| **hsa-miR-451a** | 26.53 | 26.94 | 27.86 | 24.85 | ND | ND | ND | ND | **L** |  |  |
| **hsa-miR-16-1-3p** | 27.14 | 27.02 | 26.71 | 25.72 | ND | ND | ND | ND | **L** |  |  |
| **hsa-miR-206** | 27.59 | 27.68 | 27.99 | 24.28 | ND | ND | ND | ND | **L** |  |  |
| **hsa-miR-382-5p** | ND | ND | ND | ND | 13.72 | 13.54 | 14.11 | 13.86 | **G** |  |  |
| **hsa-let-7e-5p** | ND | ND | ND | ND | 15.67 | 15.45 | 13.66 | 14.55 | **G** |  |  |
| **hsa-miR-339-5p** | ND | ND | ND | ND | 17.76 | 17.60 | 18.45 | 17.73 | **G** |  |  |
| **hsa-miR-192-5p** | ND | ND | ND | ND | 19.69 | 19.48 | 18.72 | 19.17 | **G** |  |  |
| **hsa-miR-320b** | ND | ND | ND | ND | 21.30 | 21.35 | 20.86 | 21.03 | **G** |  |  |
| **hsa-miR-454-3p** | ND | ND | ND | ND | 21.63 | 23.65 | 20.44 | 20.02 | **G** |  |  |
| **hsa-miR-154-3p** | ND | ND | ND | ND | 23.62 | 24.27 | 23.48 | 24.84 | **G** |  |  |
| **hsa-miR-411-3p** | ND | ND | ND | ND | 24.99 | 24.53 | 25.85 | 25.62 | **G** |  |  |
| **hsa-miR-590-3p** | ND | ND | ND | ND | 26.77 | 25.86 | 24.57 | 25.90 | **G** |  |  |
| **hsa-miR-23a-5p** | ND | ND | ND | ND | 26.84 | 25.29 | 26.86 | 24.94 | **G** |  |  |

**L stands for Lost and G for Gained with inflammatory priming. ND stands for not detected or C_RT_ higher than 28.**
