# Supplementary material for: Inflammatory priming enhances mesenchymal stromal cell secretome potential as a clinical product for regenerative medicine approaches through secreted factors and EV-miRNAs: the example of joint disease
Source: Stem Cell Res Ther. 2020 Apr 28;11:165. doi: 10.1186/s13287-020-01677-9 (PMC7189600; doi:10.1186/s13287-020-01677-9)
Supplement: Supplementary file 4 — Additional file 4: Table 4. Potential BP targets based on in silico predicted miRNA-mRNA interactions. [file 13287_2020_1677_MOESM4_ESM.docx]

| **63 shared miRNAs (EVs + iEVs)** |  |  |  |
| --- | --- | --- | --- |
| **GO Category** | **p-value** | **#genes** | **#miRNAs** |
| **cellular nitrogen compound metabolic process** | **1.8E-157** | **2484** | **62** |
| **biosynthetic process** | **7.0E-109** | **2088** | **62** |
| **cellular protein modification process** | **3.1E-95** | **1304** | **60** |
| **small molecule metabolic process** | **1.6E-58** | **1187** | **61** |
| **symbiosis** | **7.3E-43** | **299** | **57** |
| **catabolic process** | **2.8E-40** | **964** | **62** |
| **cellular component assembly** | **3.6E-33** | **665** | **60** |
| **response to stress** | **4.4E-24** | **1062** | **61** |
| **macromolecular complex assembly** | **2.2E-23** | **441** | **59** |
| **biological_process** | **3.5E-23** | **7745** | **62** |
| **cell death** | **7.2E-22** | **464** | **58** |
| **nucleobase-containing compound catabolic process** | **1.2E-19** | **430** | **58** |
| **membrane organization** | **1.5E-19** | **291** | **57** |
| **cell-cell signaling** | **1.7E-18** | **339** | **55** |
| **protein complex assembly** | **2.2E-13** | **360** | **58** |
| **cell junction organization** | **2.8E-12** | **90** | **55** |
| **immune system process** | **5.4E-12** | **717** | **60** |
| **cell motility** | **3.8E-10** | **275** | **57** |
| **vesicle-mediated transport** | **3.0E-08** | **479** | **57** |
| **generation of precursor metabolites and energy** | **7.0E-08** | **154** | **55** |
| **DNA metabolic process** | **1.9E-07** | **337** | **57** |
| **homeostatic process** | **2.3E-07** | **367** | **58** |
| **sulfur compound metabolic process** | **1.0E-06** | **130** | **51** |
| **extracellular matrix organization** | **2.3E-04** | **162** | **54** |
|  |  |  |  |
| **hsa-miR-320a-3p/132-3p (only EVs)** |  |  |  |
| **GO Category** | **p-value** | **#genes** | **#miRNAs** |
| **cellular nitrogen compound metabolic process** | **7.1E-58** | **530** | **2** |
| **biosynthetic process** | **5.5E-38** | **433** | **2** |
| **cellular protein modification process** | **2.0E-28** | **269** | **2** |
| **symbiosis** | **2.4E-14** | **69** | **2** |
| **response to stress** | **1.6E-12** | **224** | **2** |
| **small molecule metabolic process** | **2.3E-11** | **213** | **2** |
| **cellular component assembly** | **2.9E-10** | **133** | **2** |
| **cell death** | **3.8E-10** | **103** | **2** |
| **catabolic process** | **7.9E-10** | **180** | **2** |
| **membrane organization** | **1.1E-09** | **69** | **2** |
| **macromolecular complex assembly** | **8.8E-09** | **93** | **2** |
| **nucleobase-containing compound catabolic process** | **9.4E-07** | **87** | **2** |
| **protein complex assembly** | **3.3E-06** | **77** | **2** |
| **mRNA processing** | **7.3E-06** | **64** | **2** |
| **DNA metabolic process** | **3.5E-05** | **75** | **2** |
| **immune system process** | **1.4E-04** | **137** | **2** |
| **cell junction organization** | **2.7E-04** | **20** | **2** |
| **histone binding** | **7.4E-04** | **25** | **2** |
|  |  |  |  |
| **hsa-miR-382-5p/let-7e-5p (only iEVs)** |  |  |  |
| **GO Category** | **p-value** | **#genes** | **#miRNAs** |
| **cellular nitrogen compound metabolic process** | **1.8E-76** | **615** | **2** |
| **biosynthetic process** | **5.8E-60** | **526** | **2** |
| **cellular protein modification process** | **1.3E-57** | **359** | **2** |
| **symbiosis** | **1.7E-28** | **98** | **2** |
| **catabolic process** | **2.4E-24** | **245** | **2** |
| **small molecule metabolic process** | **1.8E-21** | **268** | **2** |
| **cellular component assembly** | **1.1E-17** | **167** | **2** |
| **macromolecular complex assembly** | **6.8E-17** | **123** | **2** |
| **response to stress** | **9.4E-16** | **255** | **2** |
| **membrane organization** | **2.4E-14** | **85** | **2** |
| **nucleobase-containing compound catabolic process** | **3.9E-13** | **114** | **2** |
| **DNA metabolic process** | **8.1E-12** | **103** | **2** |
| **cell death** | **1.3E-11** | **115** | **2** |
| **protein complex assembly** | **7.0E-09** | **92** | **2** |
| **ribonucleoprotein complex assembly** | **6.5E-07** | **27** | **2** |
| **immune system process** | **8.7E-06** | **156** | **2** |
| **cell junction organization** | **1.3E-05** | **24** | **2** |
| **cell cycle** | **1.4E-05** | **107** | **2** |
| **sulfur compound metabolic process** | **1.5E-05** | **37** | **2** |
| **carbohydrate metabolic process** | **2.2E-05** | **108** | **2** |
| **generation of precursor metabolites and energy** | **4.8E-05** | **40** | **2** |
| **mRNA processing** | **4.3E-04** | **62** | **2** |
| **cytoskeleton organization** | **4.9E-04** | **71** | **2** |
| **microtubule organizing center** | **9.1E-04** | **50** | **2** |

Legend: Blue GO for terms only present in “shared” list, Green GO for terms only present in “hsa-miR-320a-3p/132-3p” list, Red GO for terms only present in “hsa-miR-382-5p/let-7e-5p” list, Grey GO for terms present in “hsa-miR-320a-3p/132-3p and hsa-miR-382-5p/let-7e-5p” lists, Yellow GO for terms present in “shared and hsa-miR-382-5p/let-7e-5p” lists. No background for terms present in all three lists.
