# Supplementary material for: Inflammatory priming enhances mesenchymal stromal cell secretome potential as a clinical product for regenerative medicine approaches through secreted factors and EV-miRNAs: the example of joint disease
Source: Stem Cell Res Ther. 2020 Apr 28;11:165. doi: 10.1186/s13287-020-01677-9 (PMC7189600; doi:10.1186/s13287-020-01677-9)
Supplement: Supplementary file 5 — Additional file 5: Table 5. Ingenuity Pathway Analysis on experimentally verified miRNA-mRNA interactions. [file 13287_2020_1677_MOESM5_ESM.docx]

| **Osteoarthritis Pathway** | **Inhibition of matrix metalloproteases** | **Inflammasome** | **Chemokine signalling** | **Altered T and B cell signalling** | **Role of Macrophages, fibroblasts and endotehlial cells** |
| --- | --- | --- | --- | --- | --- |
| **hsa-let-7a-5p** |  | **hsa-let-7a-5p** | **hsa-let-7a-5p** | **hsa-let-7a-5p** | **hsa-let-7a-5p** |
|  |  |  |  |  | **hsa-let-7c-5p** |
| **hsa-miR-16-5p** | **hsa-miR-16-5p** | **hsa-miR-16-5p** | **hsa-miR-16-5p** |  | **hsa-miR-16-5p** |
| **hsa-miR-19b-3p** |  |  |  |  | **hsa-miR-19b-3p** |
| **hsa-miR-21-5p** | **hsa-miR-21-5p** |  |  | **hsa-miR-21-5p** | **hsa-miR-21-5p** |
| **hsa-miR-24-3p** |  |  | **hsa-miR-24-3p** |  | **hsa-miR-24-3p** |
| **hsa-miR-26a-5p** |  |  |  |  | **hsa-miR-26a-5p** |
|  |  |  |  |  | **hsa-miR-26b-5p** |
|  |  |  |  |  | **hsa-miR-27a-3p** |
| **hsa-miR-27b-3p** | **hsa-miR-27b-3p** |  |  |  | **hsa-miR-27b-3p** |
| **hsa-miR-29b-3p** |  |  |  |  | **hsa-miR-29b-3p** |
| **hsa-miR-30a-5p** |  |  | **hsa-miR-30a-5p** |  | **hsa-miR-30a-5p** |
| **hsa-miR-31-5p** |  |  |  |  | **hsa-miR-31-5p** |
| **hsa-miR-34a-5p** |  |  | **hsa-miR-34a-5p** |  |  |
| **hsa-miR-92a-3p** |  |  |  |  | **hsa-miR-34a-5p** |
| **hsa-miR-99b-5p** |  |  |  |  |  |
| **hsa-miR-106a-5p** | **hsa-miR-106a-5p** |  |  | **hsa-miR-106a-5p** | **hsa-miR-106a-5p** |
| **hsa-miR-125b-5p** |  | **hsa-miR-125b-5p** | **hsa-miR-125b-5p** | **hsa-miR-125b-5p** | **hsa-miR-125b-5p** |
| **hsa-miR-127-5p** |  |  |  |  |  |
| **hsa-miR-130a-3p** |  |  |  | **hsa-miR-130a-3p** | **hsa-miR-130a-3p** |
| **hsa-miR-138-5p** |  |  |  |  |  |
| **hsa-miR-145-5p** | **hsa-miR-145-5p** |  |  |  | **hsa-miR-145-5p** |
| **hsa-miR-146b-5p** | **hsa-miR-146b-5p** | **hsa-miR-146b-5p** | **hsa-miR-146b-5p** | **hsa-miR-146b-5p** | **hsa-miR-146b-5p** |
| **hsa-miR-149-5p** |  |  | **hsa-miR-149-5p** |  | **hsa-miR-149-5p** |
| **hsa-miR-181a-5p** | **hsa-miR-181a-5p** |  | **hsa-miR-181a-5p** | **hsa-miR-181a-5p** | **hsa-miR-181a-5p** |
|  |  |  |  | **hsa-miR-191-5p** | **hsa-miR-191-5p** |
| **hsa-miR-193a-5p** |  |  | **hsa-miR-193a-5p** | **hsa-miR-193a-5p** | **hsa-miR-193a-5p** |
| **hsa-miR-193b-3p** |  |  |  |  | **hsa-miR-193b-3p** |
| **hsa-miR-199a-3p** |  |  |  |  | **hsa-miR-199a-3p** |
| **hsa-miR-210-3p** |  |  |  |  | **hsa-miR-210-3p** |
| **hsa-miR-214-3p** |  |  |  |  | **hsa-miR-214-3p** |
| **hsa-miR-218-5p** |  |  | **hsa-miR-218-5p** |  | **hsa-miR-218-5p** |
| **hsa-miR-222-3p** | **hsa-miR-222-3p** |  | **hsa-miR-222-3p** |  | **hsa-miR-222-3p** |
|  |  |  |  |  | **hsa-miR-296-5p** |
| **hsa-miR-320-3p** |  |  |  |  |  |
| **hsa-miR-483-5p** |  |  |  |  |  |
